# Differentiation potential of Pluripotent Stem Cells correlates to the level of CHD7

**DOI:** 10.1038/s41598-017-18439-y

**Published:** 2018-01-10

**Authors:** Takako Yamamoto, Chiemi Takenaka, Yusuke Yoda, Yasuhiro Oshima, Kenichi Kagawa, Hiroshi Miyajima, Tetsuji Sasaki, Shin Kawamata

**Affiliations:** 1 Foundation Biomedical Research and Innovation (FBRI), Research and Development Center for Cell Therapy 2-2 Minatojima-Minamimachi, Chuo-ku, Kobe, 650-0047 Japan; 2 Riken Center for Developmental Biology (CDB) 2-2-3 Minatojima Minamimachi, Chuo-ku, Kobe, 650-0047 Japan; 3 Tokyo Electron Ltd. Innovative Technology Planning Dept. Akasaka Biz Tower, 5-3-1 Akasaka, Minato-Ku, Tokyo, 107-6325 Japan; 4 Tokyo Electron Europe Ltd. Unite G1, Stevenage Bioscience Catalyst Gunnel Wood Road, Stevenage, Hertfordshire, SG1 2FX United Kingdom; 5 Product Development Division, Kyokuto Pharmaceutical Industrial Co., Ltd., 3333-26, Aza-Asayama, Kamitezuna Takahagi-shi, Ibaraki, 318-0004 Japan

## Abstract

Embryonic Stem Cells (ESC) possesses two distinct features; self-renewal and the potential to differentiate. Here we show the differentiation potential and growth rate of ESC correlates positively with the expression level of the gene encoding chromodomain helicase DNA binding protein 7 (CHD7). When ESCs are maintained in feeder-free conditions and single cell seeding, ESC KhES-1 having 4520 copies or more of *CHD7* in 5 ng total RNA show differentiation potential, but this is lost when the *CHD7* copy number is reduced in KhES-1 to less than 696 by alternative culture conditions. Introduction of si*CHD7* reduced differentiation potential and growth rate of KhES-1. Interestingly, KhES-1 underwent spontaneous differentiation when *mCHD7* was introduced and we could not obtain CHD7-overexpressing ESC in culture. These data suggest that CHD7 drives differentiation, and there is a lower limit for CHD7 to initiate differentiation and an upper limit for CHD7 if maintained in undifferentiated state, and such upper limit varies depending on culture condition. As CHD7 drives cell growth, ESC with the highest permissible CHD7 level in the given culture become dominant in a couple of passages. Thus, we can select differentiation resistance-free cell clones by optimizing the culture system using *CHD7* as an index.

## Introduction

Embryonic stem cells (ESCs) and induced pluripotent stem cells (iPSCs) are pluripotent stem cells (PSCs) that have two distinct features: the ability to proliferate in the undifferentiated state (self-renewal) and the potential to differentiate in response to differentiation stimuli. However, the potential to differentiate can only be verified by exposing PSCs to external differentiation signals. Prediction of the differentiation potential while maintaining PSCs in the undifferentiated state is particularly important for clinical applications of PSC-derived cell products^1^ because inclusion of undifferentiated cells or differentiation-resistant cells in the final PSC-derived cell product may lead to the development of tumors after transplantation. In this context, identification of markers in PSCs that can predict the resistance to differentiation would be quite useful while maintaining them in the undifferentiated state. Here, we report our analysis of this issue.

## Results

### The differentiation potential of ESCs was altered by changing culture conditions

**Figure 1:**
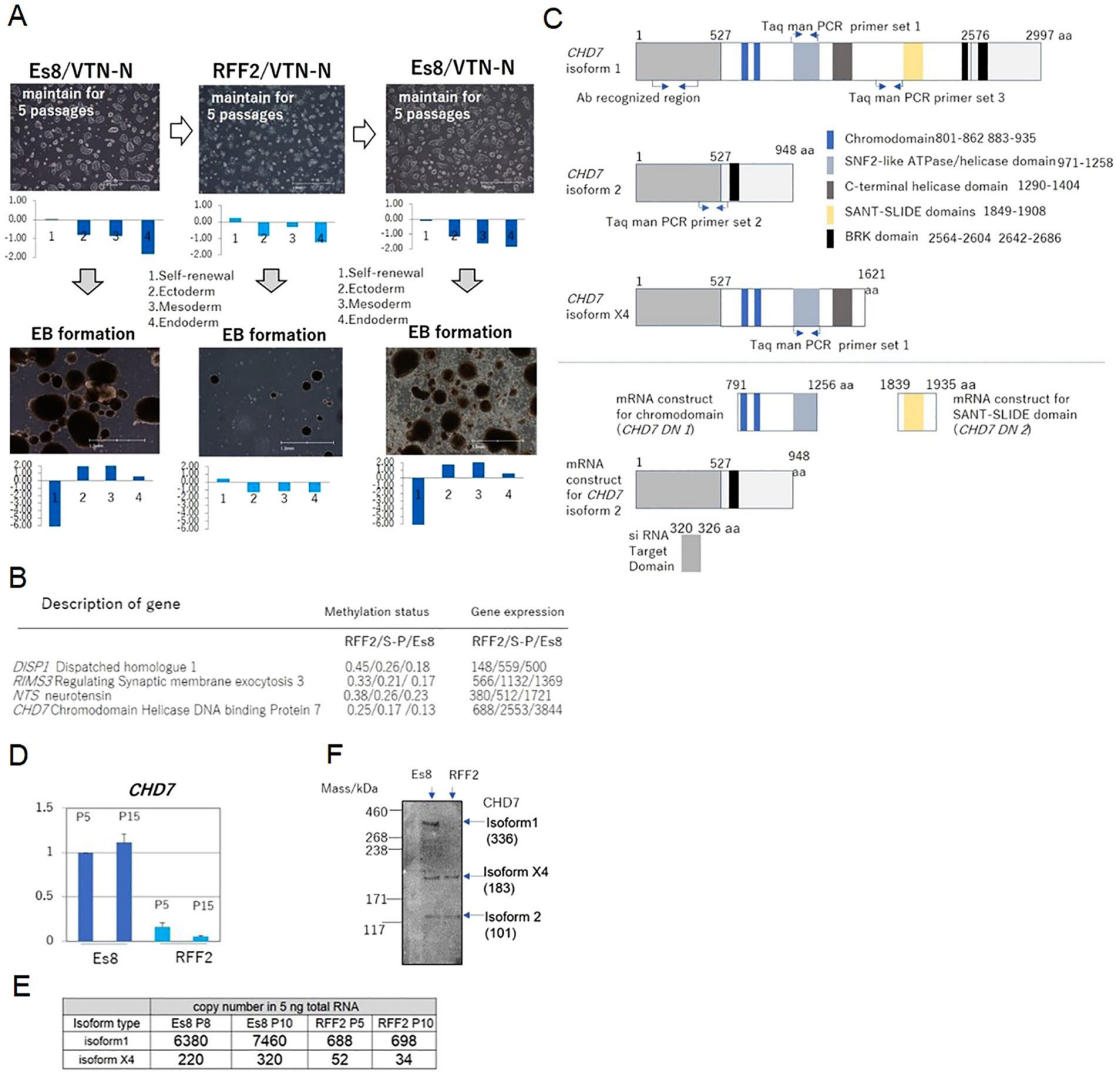
The differentiation potential of ESCs was altered by culture conditions. (**A**) KhES-1 ESCs in single-cell suspensions were seeded on VNT-N-coated dishes and cultured with Essential 8 (Es8) for 5 passages. The cells were then collected for embryoid body (EB) formation or transferred to Repro FF2 culture medium (RFF2). KhES-1 cells were cultured for 5 passages and collected for EB formation or transferred to Es8 again. KhES-1 cells were cultured for 5 passages, followed by EB formation assays. Photographs of KhES-1 cultures with Es8 or RFF2 medium (upper) at day 1 of culture; EBs at day 14 (lower) are shown. Gene expression profiles of cells in the indicated culture conditions were determined by a qRT-PCR scorecard panel and appended below the relevant photograph. Scale bar: 1.0 mm. (**B**) List of candidate genes and short description related to differentiation potential of PSCs. Value of methylation status and corresponding its gene expression in PSCs cultured with RFF2, S-P and Es8 and shown as in the order of RFF2/S-P/Es8. (**C**) Schematics of *CHD7* isoforms, location of PCR primers and antibody (upper), and *CHD7* mRNA transcripts used in this study (lower). (**D**) Gene expression of *CHD7* in KhES-1 cultures with Es8 (P5 and P15) or RFF2 medium (P5 and P15) determined by qRT-PCR. P: passage numbers. (n = 3 analytical replicates). (**E**) Copy numbers of CHD7 isoform 1 or isoform X4 determined by digital-PCR. Five ng of total RNA obtained from KhES-1 cells cultured with Es8 or RFF2 medium were used as the template, and the copy numbers of the isoforms in each RNA sample were calculated using primer set listed in Fig. 1C. (**F**) CHD7 isoforms detection by Western blotting. Total cell lysates (5.3 μg) from KhES-1 cells cultured with Es8 (P11) or RFF2 medium (P21) were applied to the indicated lanes. CHD7 isoform 1 (expected mass 336 kDa), isoform 2 (101 kDa), and isoform X4 (183 kDa) were detected with antibodies for human CHD7. The signal was visualized by a secondary antibody linked to horseradish peroxidase.

When ESC KhES-1 were cultured with Essential 8 (Es8) on recombinant human Vitronectin-N (VTN-N)-coated dishes by seeding in single cells, they proliferated in the undifferentiated state and retained the potential to differentiate, as manifested by formation of Embryoid Bodies (EBs). The morphology and gene expression profile in EBs, as assessed by a qRT-PCR scorecard panel are shown in Fig. 1A and Figure S1. However, KhES-1 lost their differentiation potential following culture with Repro FF2 (RFF2) for five passages on VTN-N after single cell seeding. Although KhES-1 proliferated in the undifferentiated state, they could not differentiate nor survive well under an EB formation culture system. Interestingly, they regained the potential to differentiate after cultivation in Es8 (Fig. 1A and Figure S1) for five passages. We also found that KhES-1 cells cultured with Stem-Partner (S-P) retained the potential to differentiate, but that this potential was lost by culturing with RFF2. Furthermore, iPSC PFX#9 cultured with Es8 and then with RFF2 on VTN-N-coated dishes showed the same results (Figure S2).

It should be also noted that KhES-1 can form EBs when they are cultured with RFF2 in cell clumps either on feeder cells or on VTN-N coated-dishes (Figure S3). Therefore, we speculated that the differentiation potential of PSCs can be defined by the culture system consisting of culture medium, dish coating material and cell seeding protocol.

To explore the epigenetic mechanism by which PSCs showed distinct differentiation potential in response to culture medium, we conducted comparative methylation status studies of PSCs using methylation bead kits to identify characteristic methylation sites related to the “loss of differentiation potential”. The number of genes methylated under RFF2 culture conditions or that with S-P/Es8 culture conditions is shown in Figure S4A. Clustering of methylation patterns in the promoter region is shown in Figure S4B. The methylation status of the promoters of major genes is shown in Figure S4C. We extracted genes by principle component analysis (PCA) on methylation status and gene expression by GeneChip analysis and identified 4 genes as candidates: Dispatched homologue 1 (*DISP1*), Regulating Synaptic membrane exocytosis 3 (*RIMS3*), neurotensin (*NTS*) and Chromodomain Helicase DNA binding Protein 7 (*CHD7*) (Fig. 1B). Among these genes, CHD7 is of specific interest since it has previously been shown to play a regulatory role in organ development. Mutations in this gene have been shown to result in CHARGE syndrome^2,3^. We found no major effect on the gene methylation status of self-renewal factors such as *NANOG*, *POU5F1*, *REX1*, *SOX2* or *p300* by changing media (Figure S4C). Expression of *CHD7* isoforms^4,5^ was determined with the set of PCR primers listed in Fig. 1C and protein expression levels of CHD7 isoforms were analyzed with antibody recognizing 5′ exons of all isoforms respectively. We detected marked difference in *CHD7* levels in KhEs-1 cultured with Es8 and RFF2 with the primer set targeted to the 3′ non-coding region of the gene (Fig. 1D). The copy numbers of isoform 1as assessed by Taq man PCR primer set (Fig. 1E) and the protein expression level of isoform 1 as detected by Western blotting differed markedly between two culture systems (Fig. 1F). As for further quantification, enzyme-linked immunosorbent assays (ELISA) were not a feasible option for quantifying each isoform because the molecular mass of the smallest isoform of CHD7, isoform 2, exceeded 101 kDa. We could not obtain synthetic proteins of this size with reliability and high purity to establish a calibration curve. We expected comparable copy numbers of isoform 2 and isoform X4, based on the result of Western blotting. However, we were unable to directly measure the copy number of isoform 2, because the Taq man primer set 2 designed to amplify upstream of aa 527 and downstream of aa 2576 of isoform 1 gave unreliable replicates. Structural analysis of CHD7 isoforms indicated that isoform 1 contains a regulatory region stretching from amino acids 527 to 2576. This region contains ATPase/DNA helicase domains, a chromosome binding domain, a DNA binding domain and a BRK domain^4,6,7^. Isoform 2 as a splicing variant of isoform 1 lacks such a regulatory region. Isoform X4, another splicing variant of isoform 1, lacks the C-terminal half of isoform 1 (Fig. 1C). No reports have yet described the functions of isoforms 2 and X4. We assumed that CHD7 isoform 1, as the major CHD7 isoform, would mediate transcriptional activity and differentiation processes in PSCs through regulatory domains, and conducted a set of experiments to address the function of isoform 1.

Our observations showed that the differentiation potential of PSCs was related to the expression of *CHD7*. We therefore examined whether KhEs-1 cultured with Es8 would lose their differentiation potential if protein expression levels of CHD7 were specifically reduced by the introduction of si*CHD7* (Fig. 1C), and whether KhES-1 cultured with RFF2 underwent differentiation when *CHD7* mRNA was introduced to increase CHD7 protein expression levels. However, we could not engineer mRNA for full-length *CHD7* isoform 1 due to the length of the coding region (2997aa). We generated and introduced *CHD7* isoform 2 (948aa) mRNA instead to KhEs-1 cultured with RFF2, and examined the functions of the helicase domains of CHD7. The function of the regulatory domains of isoform1 were examined by introducing mRNA encoding the regulatory domains (Fig. 1C) to KhES-1.

### Downregulation of *CHD7* perturbed differentiation

**Figure 2:**
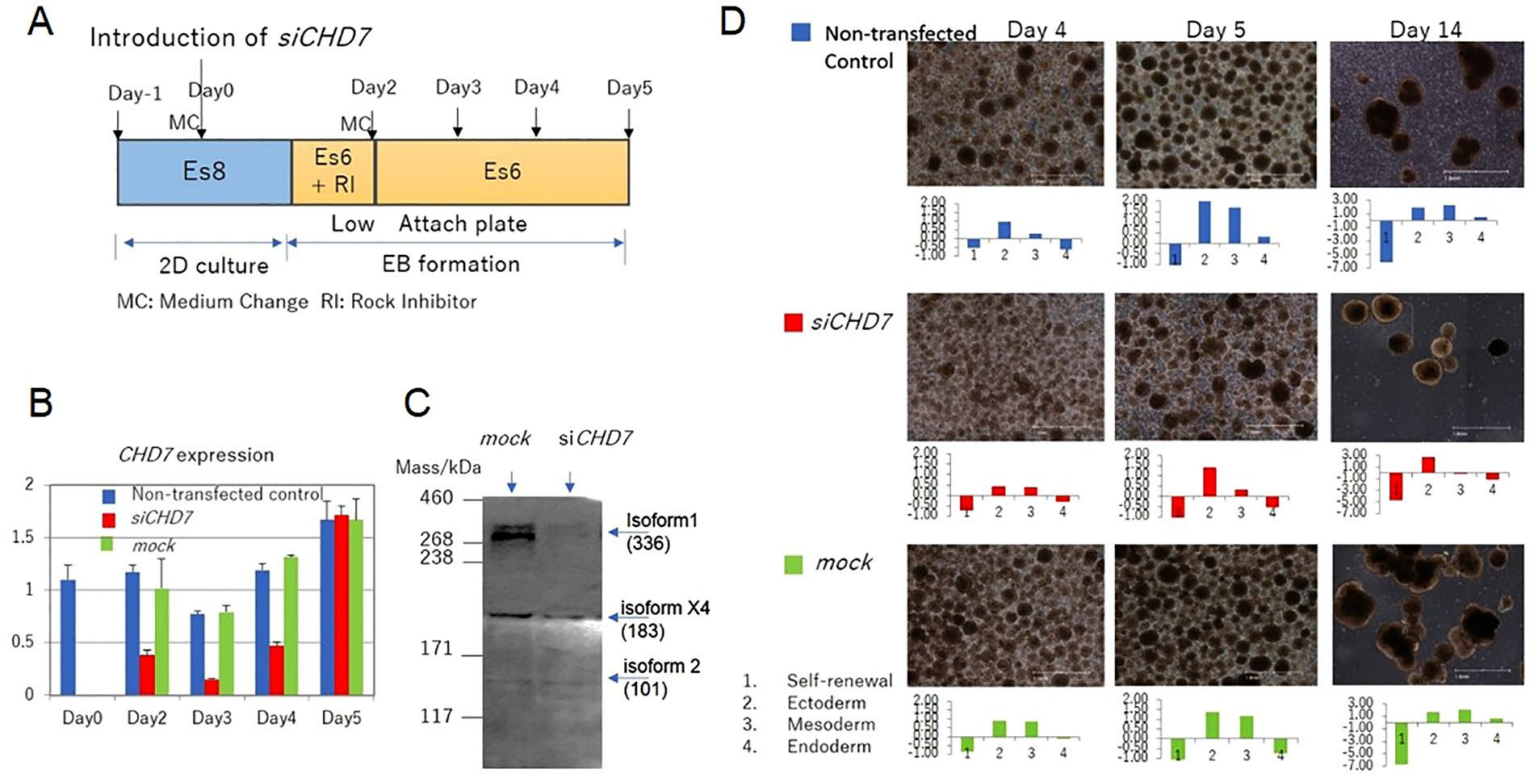
Downregulation of *CHD7* disrupted differentiation in EB formation assays. (**A**) Protocol for EB formation and *siCHD7* transfection. Cells were transfected with small double-stranded interfering RNA targeting *CHD7* (*siCHD7*) or nonspecific control siRNA (mock) on day 0. Cells were transferred to low-attachment plates 24 h after transfection with siRNA and cultured with Es6 supplemented with ROCK inhibitor (RI) for 24 h. The medium was then exchanged with fresh Es6 medium and cultured for another 72 h. The morphology of EBs and their gene expression profiles on days 4, 5 and 14 after transfection with siRNA were determined using a qRT-PCR scorecard panel. (**B**) Expression of *CHD7* in KhES-1 cells transfected with *siCHD7* or control siRNA (mock) or in non-transfected cells determined by qRT-PCR (time course sampling). Gene expression of *CHD7* was standardized according to the average *CHD7* expression in KhES-1 cells cultured with Es8 medium, which was independently measured 3 times. A representative result from 3 biological replicates. (n = 3 analytical replicates). (**C**) Expression of CHD7 in KhES-1 cells transfected with *siCHD7* or control siRNA (mock) by Western blotting (sampling at day 2). (**D**) Photographs of non-transfected EBs (upper panels) and *siCHD7*-transfected (middle panels) or control siRNA-transfected (mock, lower panels) KhES-1 cells on days 4, 5, and 14 are shown. Gene expression profiles of KhES-1 cells under the designated conditions, as determined by qRT-PCR scorecard panel, are shown below the relevant photographs. Scale bar: 1 mm. The representative datasets from 3 independent experiments are shown.

KhES-1 cells cultured with Es8 retained their potential for differentiation and formed EBs in Es6 medium. The ability of KhES-1 cells transfected with *siCHD7* to differentiate into each of the three germ layers was examined by an EB formation assay (Fig. 2A,B,C). Although downregulation of *CHD7* by introduction of *siCHD7* was transient, we observed a smaller size of EBs formed from si*CHD7*-transfected KhES-1 compared with ones from non- or mock-transfected KhES-1 at day14 (Fig. 2D) and perturbed ectoderm and mesoderm differentiation in EBs at day 4 and 5, in addition to perturbed mesoderm and endoderm differentiation at day 14 (Figure S5). The relation between the level of *CHD7* and differentiation potential of ESC manifested by gene expression profiles during EB formation assay was demonstrated by introducing various concentrations of si*CHD7* to KhES-1 (Figure S6).

PSCs can be effectively cultured with Es8 on VTN-N-coated dishes. When PSCs are cultured with nutrient-depleted Es8 medium (i.e., omission of daily medium changes), PSCs are no longer supported by the PSC culture system and undergo differentiation. Thus, culturing cells with nutrient-depleted medium provides another model for the induction of differentiation. In this context, mock-transfected KhES-1 cells cultured with nutrient-depleted Es8 medium differentiated, as demonstrated by qRT-PCR, and were not maintained in the culture. In contrast, *siCHD7*-transfected KhES-1 cells exhibited a relatively undifferentiated genetic profile and maintained in the culture (Figure S7). Thus, downregulation of *CHD7* is capable of blocking the differentiation triggered by nutrient depletion.

### Introduction of *CHD7* mRNA induced three germ layer differentiation

**Figure 3:**
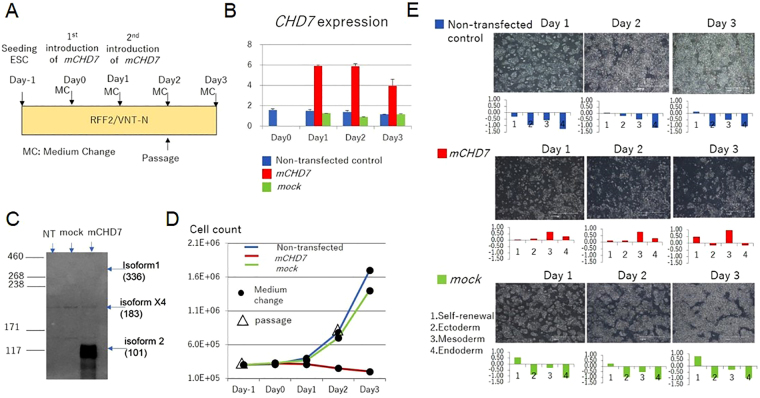
Upregulafx1tion of *CHD7* isoform 2 mRNA induced “spontaneous” differentiation in ESCs. (**A**) Protocol for cell culture and transfection with *mCHD7*. *mCHD7* or control mRNA (mock) was transfected into KhES-1 cells on day 0 and day 1 (two times). Cells were passaged on day 2, reseeded at 3 × 10^5^ cells/well in 6-well plates, and cultured another 24 h. (**B**) Expression of *CHD7* after *mCHD7* or control mRNA (*GFP*-transfected; mock) transfection (day 0) was determined by qRT-PCR (time course sampling). *CHD7* gene expression by qRT-PCR was standardized according to the average *CHD7* expression in KhES-1 cells cultured with RFF2 medium measured independently 3 times. A representative result from 3 biological replicates. (n = 3 analytical replicates). (**C**) Western blotting (sampling at day 3) of CHD7 in KhES-1 after *mCHD7* or control mRNA (*GFP*-transfected; mock) was transfected at day 0. NT: non-transfected control. (**D**) The growth of mock-transfected KhES-1 cells (green line) was comparable to that in non-transfected control cells (blue line), whereas that of *mCHD7*-transfected KhES-1 cells (red line) was dramatically suppressed on days 2 and 3. (**E**) Photographs of non-transfected (upper panels), *mCHD7*-transfected (middle panels), and mock mRNA-transfected (lower panels) KhES-1 cells cultured with RFF2 medium on days 1, 2 and 3. The gene expression profiles obtained by qRT-PCR scorecard panel are presented below the relevant photo. Scale bar: 1 mm. The representative datasets from 3 independent experiments are shown.

When mRNA encoding the *CHD7* isoform 2 (Fig. 1C) (that lacks the regulatory domain of isoform 1) was introduced into KhES-1 cells cultured with RFF2, it induced “spontaneous” differentiation in the absence of any additional differentiation stimuli. The PSC culture system employed is designed to maintain undifferentiated cells, not differentiated cells, KhES-1 cells cannot be cultured on VTN-N-coated dishes when differentiated, and the cell number of KhES-1 in RFF2 cultures was reduced as the cells differentiated (Fig. 3). Interestingly, introduction of *CHD7* isoform 2 induced simultaneous three germ layer differentiation without following the sequential differentiation process as observed by analysis of gene expression profiles (Figure S8). This observation suggested that regulatory domains present in isoform 1of CHD7 (aa527-2576), which are spliced out in isoform 2, could guide the differentiation sequence by specifying the targeted gene loci and methylation status of chromatin in heterochromatin.

**Figure 4:**
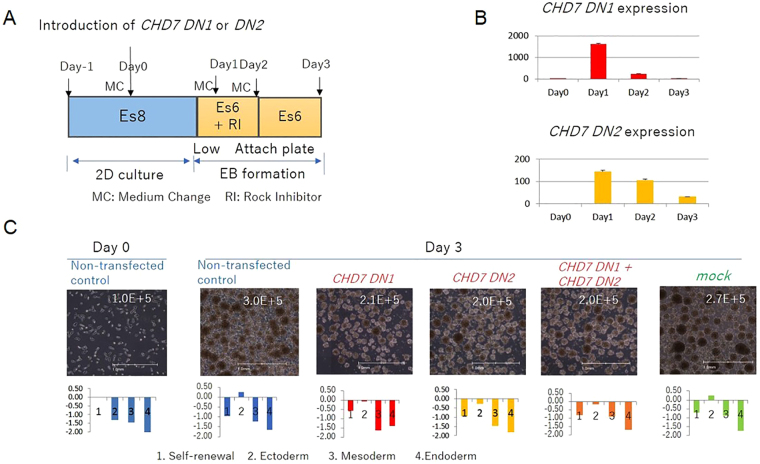
Transfection with *CHD7* dominant-negative (DN) mRNA transcripts disrupted differentiation potential and cell proliferation of ESCs. (**A**) Protocol for transfection with *CHD7* DN mRNA transcripts and EB formation assay. CHD7 DN1: transcript of chromodomain mRNA; CHD7 DN2: transcript of SANT-SLIDE domain mRNA (Figure S5B). Transcripts were transfected into KhES-1 cells on day 0, and the cells were then transferred to low-attachment plates for 24 h, followed by culture in Es6 medium with Rock Inhibitor (RI) for 24 h for EB formation. Microscopic observation of EBs and gene expression profiles by qRT-PCR scorecard panel on day 3 after transfection with DN mRNA. (**B**) *CHD7* DN 1 and *CHD7* DN2 expression levels were determined by qRT-PCR. A representative result from 3 biological replicates is shown. (n = 3 analytical replicates). (**C**) Photographs of day 3-EBs from non-transfected, *CHD7* DN1-, *CHD7* DN2-, *CHD7*-DN1 + *CHD7*-DN2-transfected, and mock mRNA-transfected KhES-1 3 days after transfection. Gene expression profiles were determined using qRT-PCR scorecard panels and are shown below the relevant photograph. The cell number on day 3 of culture in one well of a 6-well plate was scored and appended at the top right corner of the relevant image. Non-transfected KhES-1 cultured with Es8 on day 0 (left panel) was used as a control. Representative results of 3 independent experiments are shown. Scale bar: 1 mm.

To explore the function of the regulatory region of isoform 1 (aa527-2576), mRNA covering the chromodomain that recognizes a binding site for histone with a specific methylation status and the SNF2-like ATPase/helicase domain (*CHD7 DN1*) was introduced into KhES-1 cells (Figs 1C and 4A,B). Following introduction of *CHD7 DN1*, we observed a perturbation of both the differentiation potential of the cells in EB formation and the proliferation capacity of the cells. Additionally, introduction of mRNA encoding the SANT-SLID domain (*CHD7 DN2*), a putative DNA binding site of CHD7, into KhES-1 cells (Figs 1C and 4A,B) also perturbed both the differentiation potential of the cells in EB formation assays and cell proliferation (Fig. 4C, Figure S9). However, we did not observe marked synergistic suppressive effects when these transcripts were introduced together. These data indicated that blocking one of these major regulatory domains was sufficient to block the function of CHD7, and suggested that CHD7 isoform 1 would exert its function by binding to its target DNA locus specifically. In contrast CHD7 isoform 2 could bind to transcription regulatory domains and exert its helicase function in a non-site-specific fashion and trigger differentiation in a non-sequential manner when overexpressed.

Introduction of *CHD7* isoform 2 mRNA into ESCs also suggested that there may have been an upper limit for the expression of *CHD7* when ESCs were maintained in an undifferentiated state. This upper limit may vary depending on the culture conditions. Indeed, introduction of *CHD7* isoform 2 mRNA into KhES-1 cells cultured with RFF2 could generate CHD7 overexpressing KhES-1 (Fig. 3C), but these cells cannot be maintained in the culture due to “spontaneous” differentiation, and the elevated expression of *CHD7* diminished after several days (Fig. 3B). Introduction of *CHD7* isoform 2 mRNA into KhES-1 cells cultured with Es8 also would not generated KhES-1 with elevated *CHD7* expression (Figure S10). We believe that introduction of *CHD7* isoform 2 mRNA into KhES-1 cells induced “spontaneous” differentiation in KhEs-1 cells and the Es8 culture system could not support such differentiated KhES-1 cells in the culture.

### CHD7 expression levels controlled the proliferation rate of cells in the undifferentiated state

**Figure 5:**
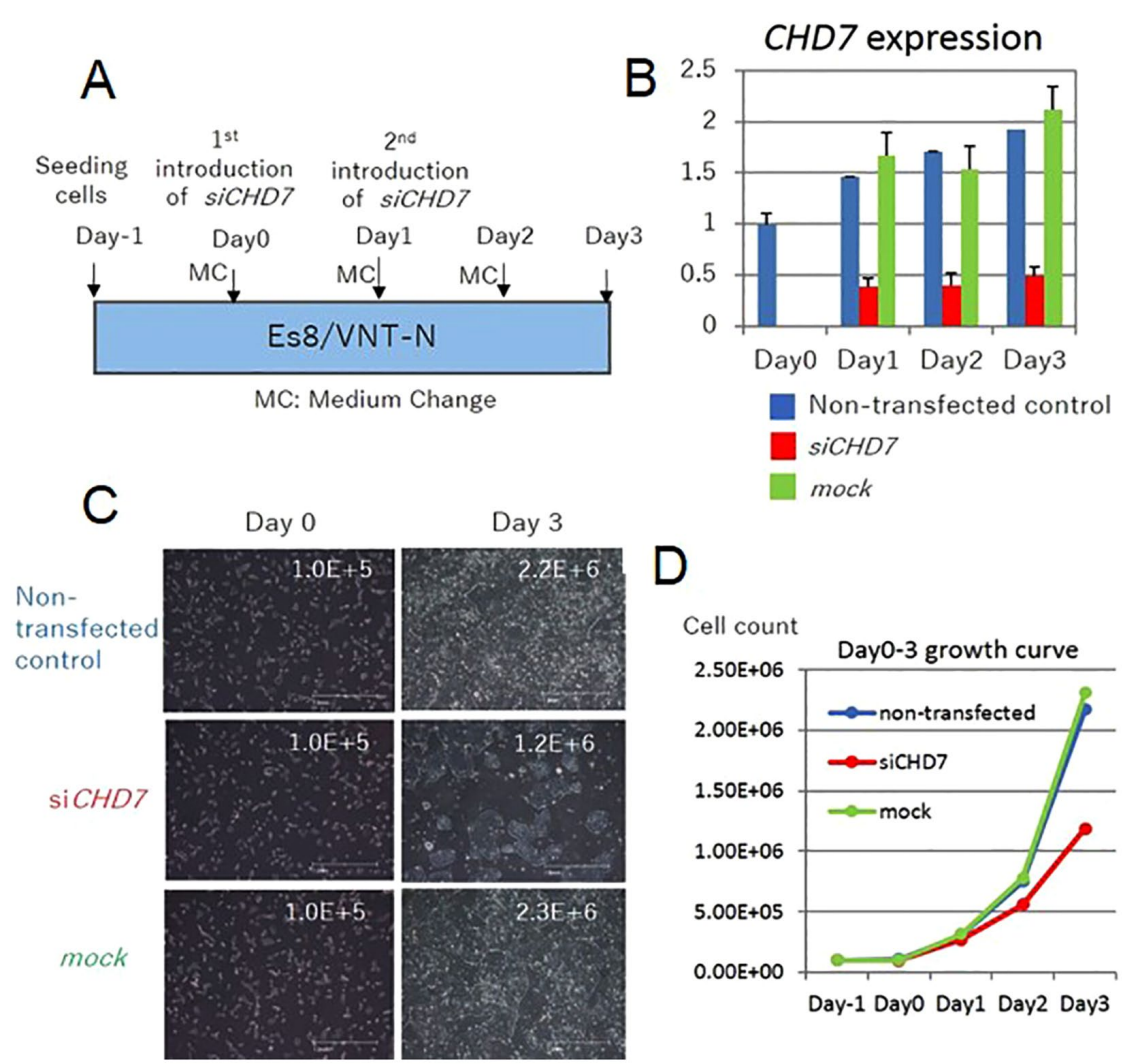
Downregulation of *CHD7* disrupted the proliferation of ESCs cultured with Es8 medium. (**A**) Protocol for *siCHD7* transfection. *siCHD7* or nonspecific control siRNA (mock) was transfected into KhES-1 cells on day 0 and day 1. Medium was changed (CM) every day. On days 0–3, cells were harvested for cell counting, and *CHD7* expression was determined by qRT-PCR. (**B**) *CHD7* gene expression in KhES-1 cells transfected with *siCHD7* or control siRNA (mock) or in non-transfected cells was determined by qRT-PCR. *CHD7* gene expression was standardized according to the average CHD7 expression in KhES-1 cells cultured with Es8 medium (independently measured 3 times). A representative result from 3 biological replicates is shown. (n = 3 analytical replicates). (**C**) Photographs of KhES-1 cells without transfection (upper panels) or with *siCHD7* (middle panels) or control siRNA transfection (mock, lower panels) on day 3. The cell number scored is appended at the upper right corner of the respective photograph. (**D**) The cell number was scored for the indicated conditions at the designated day.

An additional function of CHD7 we have observed is that it supports the proliferation of ESCs. We routinely seeded 1 × 10^5^ KhES-1 cells in one well of a 6-well plate and harvested 8 × 10^5^ cells after 3 days cultivation of the cells in Es8 medium. In contrast, the proliferation rate of KhES-1 cells in RFF2 medium was one-third that observed with Es8 medium. To examine whether CHD7 expression levels modulated the proliferation rate of ESCs in a concentration-dependent manner, we downregulated *CHD7* by introduction of *siCHD7* into KhES-1 cells, cultured the cells with Es8 medium and determined cell counts during culture (Fig. 5A,B). We found that the cell’s proliferation rate was regulated by the expression level of *CHD7* mRNA in KhES-1 cells (Fig. 5C,D). Moreover, introduction of dominant-negative forms of *CHD7* mRNA covering the chromodomain (*DN1*) and/or SANT-SLIDE domain (*DN2*) into KhES-1 cells reduced the cell proliferation rate markedly (Fig. 4), suggesting that the regulatory domain of isoform 1 (aa 527-2576) participated in the machinery mediating cell proliferation in ESCs. It is presumed that cell growth can be mediated by the complex of regulatory factors and CHD7 takes some part in it. Therefore, we could not compare the growth rate of cells by the level of CHD7 only if the cell type is different.

### CHD7 co-localized with translational suppressive factors

**Figure 6:**
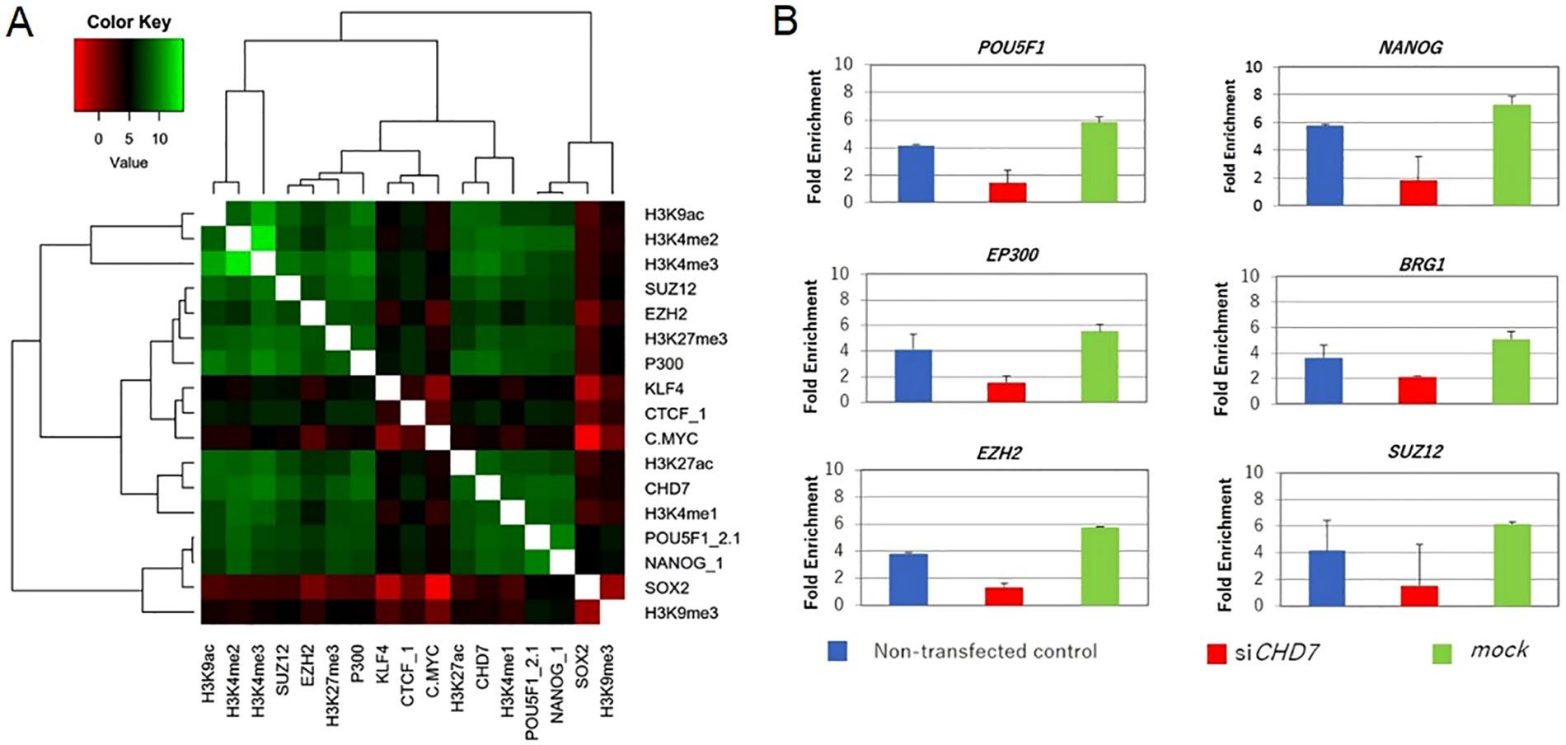
Human CHD7 co-localized with suppressive chromatin remodelers. (**A**) The binding sites for 17 factors in the map were obtained from a public ESC H1 ChIP-seq database. The odds ratio representing the correlation between binding sites for each pair of factors was calculated. Green indicates high homology between factors, and red indicates no high homology between factors. (**B**) ChIP-qPCR with anti-CHD7 antibody for *mock*- or si*CHD7*-transfected H9 cells. The *mock*- or si*CHD7* -transfected H9 cells in undifferentiated state were immune-precipitated (IP) either with anti-CHD7 antibody or control IgG. qPCR for the putative promoter region of *POU5F1*, *NANOG*, *EP300*, *EZH2*, *SUZ12* or *BRG1* were conducted for IP samples. Respective bar shows as fold enrichment of quantity of amplified target gene of interest by IP with anti-CHD7 antibody against that with control IgG in *mock*- or si*CHD7* H9 transfectants. (n = 3 analytical replicates).

In our experiments, we showed that the level of CHD7 protein was closely related to the differentiation potential of ESCs. To explore the CHD7-mediated differentiation model, we examined the possibility of co-localization of CHD7 with chromatin remodelers using a chromatin immunoprecipitation (ChIP)-seq database for human ESC H1 (WiCell). The odds ratios representing the correlation between the binding sites for each pair of factors were calculated and clustered in a heat map. Human CHD7 co-localized with POU5F1 (Oct3/4), NANOG, p300, H3K4me1, H3K4me2, H3K4me3, and H3K27ac, but not with SOX2. SOX2 may weakly co-localize with NANOG and POU5F1 to form a self-renewal factor complex in human ESCs^8^. More interestingly, human CHD7 molecules would co-localize with negative regulators that suppressed transcription, SUZ12 and EZH2 at H3K27me3-marked heterochromatin in ESC (Fig. 6A). We speculated that CHD7 needs to localize at differentiation-related gene loci in heterochromatin to initiate differentiation. CHD7 could associate with self-renewal factors in euchromatin in the undifferentiated state, but its implication seems to be limited as ESC expressing a low level of CHD7 can be maintained in the undifferentiated state. Rather, the role of CHD7 in euchromatin may be to mediate the regulation of proliferation through the association with chromatin remodelers such as BRG1, a core factor of the PBAF or BAF complex^9^. A ChIP-seq study showed BRG-1 to co-localize with NANOG, less closely with CHD7, POU5F1, H3K27ac, H3K4me1, and H3K4me2, but not with H3K9me3 or EZH2 (Figure S11), suggesting that PBAF- or BAF-complex would associate with CHD7-containing self-renewal complexes in semi-open chromatin to support cell proliferation with a similar mechanism reported previously^10,11^.

A ChIP-qPCR study with anti-CHD7 antibody was conducted to explore the molecules whose transcriptional activities are regulated by CHD7 or CHD7-mediated complex. As ChIP-seq analysis showed that CHD7 co-existed with NANONG, POU5F1, SUZ12, EZH2 and BRG1, we examined the possibility that CHD7 could regulate the transcriptional activity of these genes. The results indicate that CHD7 or a CHD7-mediated complex selectively binds to the promoter regions of these genes (Fig. 6B) and suggested that CHD7 at least partly contributes to the maintenance of the chromatin structure shown by ChIP-seq results in both euchromatin and heterochromatin loci.

### Selection of differentiation resistance-free iPSC clones by single cell seeding and monitoring the level of *CHD7*

To extend our findings, we attempted to quantify the copy numbers of *CHD7* in iPSCs: 201B7, PFX#9 or SHh#2 and ESCs: H9 or KhES-1 seeded in small cell clumps or in single cells and cultured either with Es8, S-P or chemically defined Stem-Partner ACF (S-P ACF). The copy numbers of *CHD7* in PSCs (ESC and iPSCs) cultured on feeder cells as cell clumps increased after transferring them to VTN-N-coated dishes as single cells. These observations led to the following hypothesis: PSCs exist as an epigenetically heterogenous population and individual PSC have their own fixed epigenetic status and CHD7 expression level that cannot be altered easily by changing the culture conditions. The copy number of *CHD7* in cell clumps represents a mean of the copy numbers of these heterogenous cells, and culturing the cells by single cell seeding would select the cell population that fits the given culture and have a distinct CHD7 expression level. If this hypothesis is correct, we may select differentiation resistance-free cell PSC clones with relatively high CHD7 level from bulky cell clumps by selecting the culture conditions and present direct evidence that the differentiation potential of PSCs correlates to the expression level of CHD7 in PSCs in the undifferentiated state.

**Figure 7:**
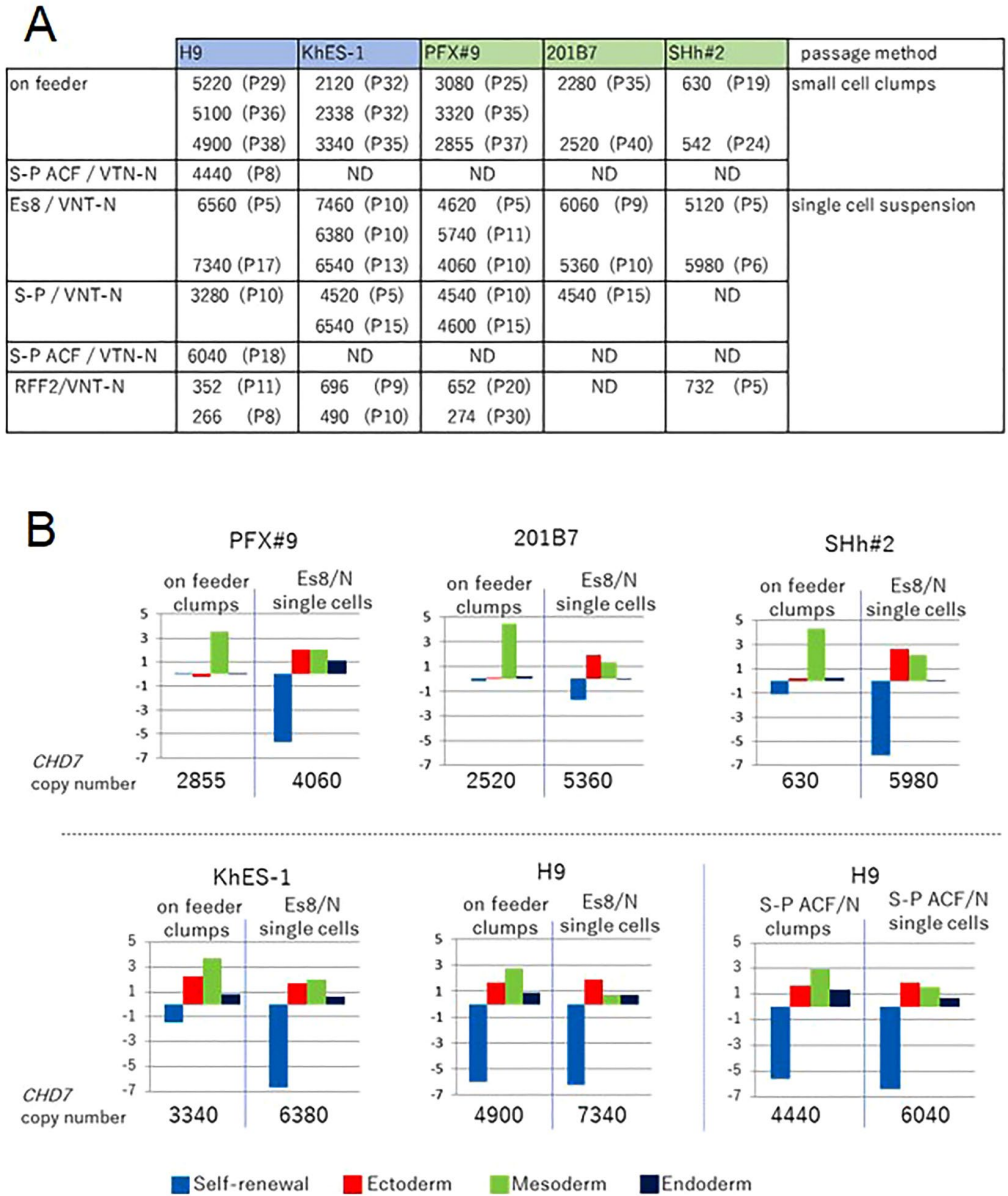
*CHD7* levels mediated the differentiation potential of PSCs. (**A**) The copy numbers of *CHD7* isoform 1 in PSCs (ESC: H9, KhES-1, iPSC: PFX#9, 201B7, SHh#2) and their passage numbers (P) are listed in the table. Cells were culture either on feeder with iPSC medium in cell clumps or with Es8, S-P, S-P ACF or RFF2 on VNT-N in single cells or in cell clumps (S-P ACF) ND: Not Determined. Cells cultured with RFF2 on VNT-N failed to demonstrate differentiation potential in EB formation assay (gray columns). (**B**) PSCs (ESC:H9, KhES-1, iPSC:PFX#9, 201B7, SHh#2) were cultured either on feeder with iPSC medium in cell clumps (on feeder cell clumps) or on VNT-N with Es8 in single cells (Es8/N single cells) or S-P ACF in cell clumps (S-P ACF/N clumps) or in single cells (S-P ACF/N single cells), and their differentiation potential was examined by the gene expression profiles of day 14-EBs derived them. The copy numbers of *CHD7* prior to EB formation assay was determined and appended beneath the relevant graph.

To explore the above hypothesis, the gene expression profiles of EBs from ESCs: H9 or KhES-1 and iPSCs: PFX#9, 201B7 or SHh#2 seeded in small cell clumps was compared with that cultured in single cell conditions with various media after measuring *CHD7* copy number in the undifferentiated state. When we examined the genetic profiles of EBs at day 14 generated from PFX#9, 201B7 or SHh#2 cultured on feeder in clumps by qRT-PCR, we observed “retarded” differentiation, meaning that the expression of self-renewal genes was not fully down regulated and the expression of differentiation lineage genes was not fully upregulated at day 14-EBs compared with those of EBs from corresponding iPSC cultured in single cell seeding conditions (Fig. 7B). However, this “retarded” differentiation was not observed in ESCs cultured either in cell clumps or on VNT-N-coated dish in single cell seeding (Fig. 7B). As an interpretation of these results, we presume that epigenetic profiles of iPSCs generated by reprogramming from somatic cells are more diverse than those of ESCs generated from uniform blastocysts that retain both proliferation and differentiation potential, and *CHD7* copy numbers would be more diverse in iPSCs than in ESC when maintained in cell clumps. iPSCs with CHD7 levels below a certain threshold may not differentiate properly, which may account for “retarded” or “perturbed” differentiation. However, this “retarded” differentiation issue of iPSCs maintained in cell clumps can be overcome if we seed iPSCs in single cells in proper feeder-free condition and select a differentiation-free iPSC population.

## Discussion

We believe this is the first report to discuss that the level of CHD7 is a key factor to evaluate the differentiation potential of PSCs while maintained in an undifferentiated state.

Hence, we need to discuss how culture conditions define the differentiation potential. Although the possibility that components in the medium alter the epigenetic status and reversibly modulate the expression level of CHD7 cannot be ruled out, this possibility is less likely. This is due to the observation that ESC cultured with RFF2 in single cell seeding conditions lost their differentiation potential, but the differentiation potential was partly recovered if ESC were cultured in cell clumps (Figure S3). The other possibility is that the culture system selects a cell population that fits for the given culture conditions, if we assume that PSCs represent a heterogenous population. The selection is made at a single cell level by growth advantage of cells, consequently cells with a higher expression level of CHD7 become dominant after several passages (Fig. 5). The CHD7 level correlates to the differentiation potential (Fig. 3) and there is an upper limit for the level of CHD7 if the cells are maintained in an undifferentiated state (Figure S10). Therefore, the cell population consisting of only the highest permissible CHD7 level becomes dominant in the given culture and cells retain the differentiation potential if the CHD7 level is above the differentiation threshold (Fig. 1D,E,F). The results in Fig. 7B are consistent with this possibility.

It was of interest to observe that the cell culture medium could determine the exact level of CHD7 in PSCs. Mammalian cells are maintained with a balance between glycolysis and oxidative phosphorylation^12,13^. In the undifferentiated state, culture media for PSCs must contain high levels of glucose to support the glycolytic pathway in PSCs^14^. RFF2 medium contains moderately high glucose (2.52 g/L) and high concentrations of protein (total protein estimated at 23 mg/mL) with a variety of amino acids. Cell Culture media such as RFF2 can be used for maintenance of PSCs in cell clumps on feeder cells or when cultures are seeded as single cells on Matrigel. However, RFF2 media is not a fully supportive media for the cell culture on VTN-N in single cells seeding condition. Indeed, a considerable portion of PSCs cultured with RFF2 died or differentiated on VNT-N-coated dishes when they were transferred from Es8 to RFF2 medium. PSCs cultured with RFF2 in this condition could not have expressed a high level of CHD7 that drives differentiation if cells are to remain in the culture in the undifferentiated state. Consequently, PSC populations having markedly low levels of CHD7 can be selected in RFF2 culture at the expense of losing differentiation potential. In contrast, Es8 medium contains high glucose levels (3.1 g/L) and a limited amount of amino acids that is designed to support PSC proliferation via active glycolytic pathway selectively in both feeder-free and single-cell seeding conditions. This culture system allows the establishment of robust self-renewal circuitry and thus allows cells to have a diverse range of CHD7 expression levels, including relatively high levels of CHD7. And again, PSC cell populations with the highest permissible level of CHD7 become dominant after several passages. It is worth noting that the selection of cell populations by culture condition might not mean the extinction of a minor population that is not suited for a given culture condition. That is the theory of equilibrium systems in population biology. By maintaining a wide epigenetic status range of PSC repertoires, a minor population can become dominant in other culture systems, which might best explain why the level of CHD7 in PSC can shift reversibly and in a timely manner just by changing culture conditions.

All together, we believe that the determination of the *CHD7* copy number in PSC is a useful index to predict differentiation potential and evaluate the usability of the culture system. Further, we can easily establish differentiation-free PSC populations in a couple of passages when seeded in single cells by the culture condition using *CHD7* copy number as an index. We believe further molecular study of related chromatin remodelers will proved further insight into the mechanism by which CHD7may regulate proliferation and differentiation in PSCs.

### Experimental Procedures

All the experiments conducted in this study used human ESCs and human iPSCs. The work was reviewed and approved by the ethical committee of the Foundation for Biomedical Research and Innovation (FBRI). This study does not contain any studies with human participants nor establishment of new iPSC cell line from human tissue.

### Cell culture

Human ESC lines KhES-1^15^ (Riken BRC) and H9^16^ (WiCell), and the human iPSC lines PFX#9^17^, 201B7^18^ (Riken BRC) and SHh#2^17^ were cultured with hPSCs culture media on mitomycin C-treated SNL76/7 cells (SIM strain embryonic fibroblast, European Collection of Cell Culture) or with chemical defined medium Essential 8^19^ (Es8, Thermo Fisher Scientific), serum-free medium Stem-Partner^20^ (S-P, Kyokuto Pharma), chemical defined medium Stem-Partner ACF (S-P ACF, Kyokuto Pharma) or xeno-free Repro FF2 medium (ReproCELL) on recombinant human Vitronectin-N (Thermo Fisher Scientific)-coated dishes. Cells were passaged in clumps using Gentle Cell Dissociation Reagent (GCDR; Stem Cell Technologies) and split at a ratio of 1:3 for iPSCs or 1:3.5 for ESCs. Alternatively, cells were passaged by seeding a single-cell suspension using TrypLE Select (Thermo Fisher Scientific). Cells in single-cell suspensions were seeded at 1 × 10^5^ cells/well in 6-well plates when cultured with Es8 medium and 3 × 10^5^ cells/well with RFF2 medium. Cells were cultured in an atmosphere containing 5% CO_2_ in an incubator (MCO-19AIC; Panasonic) at 37 °C and harvested at day 3 for passaging. Karyotypes of KhES-1, H9, and PFX#9 cells were examined by multicolor fluorescence *in situ* hybridization every 5 passages and by G-banding every 10 passages; PSCs with a normal karyotype were used in this study.

### siRNA reagents and transfection

All reagents were purchased from Thermo Fisher Scientific, unless otherwise specified. Silencer Select Pre-designed human *CHD7* siRNA (cat. 4392420; ID: s31142) and Silencer Select Negative Control No. 1 (cat. 4404021) were used for *siCHD7* or control siRNA (mock) transfection experiments, respectively. Cells were transfected as follows: ESCs (1 × 10^5^) were seeded in each well of VTN-N-coated 6-well plates and cultured with 4 mL Es8, S-P or RFF2 medium. The following day, the medium was changed, and *siCHD7* or control siRNA was transfected into the cells with Lipofectamine RNAiMAX in accordance with the manufacturer’s instructions. Briefly, cocktail A (4 µL Lipofectamine RNAiMAX Reagent and 150 µL Opti-MEM Medium) was mixed with cocktail B (1 µL of 50 µM *siCHD7* [50 pmol] or control siRNA [50 pmol] and 150 µL Opti-MEM Medium), and the mixture was then incubated for 5 min at room temperature. The mixed cocktail (240 µL) was used for transfection of ESCs with *siCHD7* (final 10 nM) or control siRNA (final 10 nM), and cells were incubated for 24 or 48 h. The transduction efficiency of the reagent was assessed by qRT-PCT for detection of *CHD7* mRNA in the transfected cells at the designated time points.

### Synthetic mRNA reagents and transfection

The T7 promoter and T7 terminator were fused into the 5′ and 3′ coding DNA sequences for *CHD7* isoform 2 (NM_001316690.1), respectively and cloned into the pMX vector. Synthetic mRNA for *CHD7* isoform 2 was then generated using an mMESSAGE mMACHINE T7 ULTRA Transcription Kit after digesting the pMX construct with SfiI. Synthetic mRNA covering both chromodomain and SNF2-like ATPase/helicase domain (NM_017780) was generated by the same method. The region of the SANT-SLIDE domain in *CHD7* was determined based on the homology with the published *CHD1* sequence^6^, and synthetic mRNA was generated in the same manner. The quantity of the resulting mRNAs was measured with an ND-1000 (Nano Drop). mRNA for enhanced green fluorescent protein (eGFP) obtained from the same vector backbone was used as the transfection control (control mRNA, mock).

### mRNA transfection

ESCs (3 × 10^5^) were seeded in each well of a VTN-N-coated 6-well plate with RFF2 supplemented with 5 ng/mL fibroblast growth factor 2 (FGF2; Peprotech). *mCHD7* or control mRNA (mock) was transfected into the cells with Lipofectamine Messenger MAX in accordance with the manufacturer’s instructions. Briefly, cocktail A (3.75 µL Lipofectamine Messenger MAX transfection reagent and 125 µL Opti-MEM Medium) was incubated for 10 min at room temperature. Then, cocktail B (2.5 µg *mCHD7* or control mRNA and 125 µL Opti-MEM Medium) was prepared and mixed with cocktail A, followed by a 5-min incubation at room temperature. The mixed cocktail (240 μL) was added to 4 mL RFF2 supplemented with 5 ng/mL FGF2, and cells were cultured with this medium for 24 h at 37 °C. The transduction efficiency of the reagent was assessed by determining the expression of *CHD7* mRNA by qRT-PCR in the transfected cells at the designated time points.

### Methylation and GeneChip analysis

The methylation state of cultured ESCs or iPSCs was determined with the Infinium HumanMethylation450 BeadChip (Illumina). The methylation pattern in the promoter region was hierarchically clustered using Cluster 3.0 and visualized in Java Tree View. The methylation status of the respective genes in the promoter regions was assessed by a comparison with the gene expression signal obtained by GeneChip (Human Genome U133 Plus 2.0 Array; Affymetrix) array data to extract the candidate genes.

### EB formation assays after transfection with *siCHD7*

Cells cultured on VTN-N-coated wells with 4 mL Es8 medium were washed once with PBS (−) 24 h after transfection with either *siCHD7* or control siRNA (mock). Cells were then removed with a cell scraper (Iwaki), dissociated by pipetting, transferred into a low-attachment 6-well plate (Corning), and cultured with Es6 medium with 10 μM RI for 1 day and with Es6 medium alone for 13 days for EB formation. Medium was changed every 2 days. The numbers and morphologies of EBs were observed microscopically (Olympus IX71; Olympus). Gene expression for *CHD7* was determined by qRT-PCR TaqMan Scorecard Panel (A15870) and gene expression profiles were determined by using a qRT-PCR device (QuantStudio 12 K Flex). Primers for CHD7 are listed in Figure S13.

### EB formation assays after transfection with *mCHD7*

Cells cultured on VTN-N-coated wells and ReproFF2 supplemented with 5 ng/mL FGF2 were washed once with PBS (−) 24 h after introduction of either *mCHD7* or control mRNA. Then cells were removed with a cell scraper (Iwaki), dissociated by pipetting, transferred into a low attachment 6-well plate (Corning) and cultured with RFF2 without FGF2 with 10 µM ROCK inhibitor (Y-27632, Wako) for 1 day and RFF2 without FGF2 for subsequent days for EB formation. Medium was changed every day. The number and morphology of EBs were observed microscopically (Olympus IX71, Olympus). Gene expression for *CHD7* was determined by qRT-PCR and gene expression profiles were determined by TaqMan Scorecard Panel (A15870) using a qRT-PCR device (QuantStudio 12 K Flex).

### Generation of a co-localization heat map by *in silico* ChIP-seq study

ChIP-Seq public data from GEO were downloaded and assembled into one list. Odds ratios from Fisher’s exact tests, representing the correlations between binding site sequences for each pair of factors, were calculated. Molecules downloaded from GEO human HES H1 are shown in Figure S12. Odds ratios were organized in a matrix, hierarchically clustered using Cluster 3.0, and visualized in Java Tree View.

### ChIP-qPCR

ChIP-qPCR was performed in accordance with the manual for SimpleCHIP Plus Enzymatic Chromatin IP Kit (Cell Signaling Technology #9004). In brief, cell lysates from 8 × 10^6^ H9 cells (non-transfected control), *mock*- or si*CHD7*-introduced H9 cells cultured with Es8 on VTN-N coated dish were sonicated with QSONICA (Waken Bitech) after a protein-DNA crosslinking step. Samples were then subjected to immune-precipitation either with normal rabbit IgG antibody (CST #2729) or anti-human CHD7 antibody (ab #176807). DNA from precipitates were purified and used as templates for qPCR with primers designed to cover putative transcription starting site (TSS) located 1 kb upstream of 1^st^ exon of *POU5F1, NANOG, EP300, EZH2, SUZ12* or *BRG1*. RT2 SYBR Green qPCR Master Mix(Qiagen), Primer Epitect ChIP qPCR Primer (QIAGEN) and StepOnePlus (Thermo Fisher Scientific) were used for PCR step. Primers used for this assay is listed in Figure S13.

### qRT-PCR by scorecard panel and determination of *CHD7* transcription (copy numbers)

qRT-PCR for 96 genes were performed with Taqman hPSC Scorecard panel (Thermo Fisher) in accordance with the instruction manual. Interpretation of self-renewal, ectoderm, mesoderm, endoderm lineage differentiation is made base on the publication (2011, 144, 4390-452, Cell).

The copy numbers of CHD7 transcripts were determined by droplet digital PCR (Bio-Rad Laboratories). Briefly, cDNA was generated from 5 ng total RNA extracted from KhES-1 cells cultured with Es8 or RFF2 medium using TaqMan Gene Expression Assays (Hs00215010_m1; Thermo Fisher). The PCR mixture was loaded into a Bio-Rad QX-100 emulsification device, and droplets were formed following the manufacturer’s instructions. cDNA was amplified separately in an Applied Biosystems GeneAmp 9700 Thermocycler. Each reaction consisted of a 20-µL solution containing 10 µL ddPCR Probe Supermix, 1000 nM primers, 250 nM probe, and template cDNA with the following cycling conditions: 10 min at 95 °C, 40 cycles of 30 s denaturation at 94 °C and 60 s of annealing/extension at 53 °C and a final 10 min at 98 °C. After cycling, raw fluorescence data for each well were exported from the manufacturer’s software (Bio-Rad QuantaSoft v. 1.2) for analysis.

### Western Blotting for detection of CHD7

Cell lysates for Western blotting were prepared 72 h after seeding. Proteins were extracted using Complete Lysis-M (Roche), supplemented with protease inhibitor cocktail tablets (Complete Mini; Roche). Polyclonal sheep IgG anti-CHD7 primary antibodies (AF7350; R&D); donkey anti-sheep IgG (H + L) secondary antibodies conjugated with horseradish peroxidase (A16047; Thermo Fisher) were used for blotting. The signal was detected with Chemi-Lumi One Super reagents (Nacalai Tesque). Total protein was measured with a bicinchoninic acid total protein assay kit (Nacalai Tesque) prior to application to the lane.

## Electronic supplementary material

Supplementary Information
