# Supplementary material for: Differentiation potential of Pluripotent Stem Cells correlates to the level of CHD7
Source: Sci Rep. 2018 Jan 10;8:241. doi: 10.1038/s41598-017-18439-y (PMC5762636; doi:10.1038/s41598-017-18439-y)
Supplement: Supplementary file 1 — Supplementary Information [file 41598_2017_18439_MOESM1_ESM.pdf]

Supplementary Information

Differentiation potential of Pluripotent Stem Cells correlates to the level of CHD7

Takako Yamamoto, Chiemi Takenaka, Yusuke Yoda, Yasuhiro Oshima, Kenichi Kagawa, Hiroshi Miyajima, Tetsuji Sasaki, Shin Kawamata

KhES-1 Score card 96 Genes

Target Name	Category	undiff Es8	EBs Es8	undiff RFF2	EBs RFF2	undiff Es8 2nd	EBs Es8 2nd
CDH9	Ectoderm	0.08	0.76	0.02	0.04	0.23	0.92
COL2A1	Ectoderm	1.17	2.46	0.31	0.29	3.31	2.15
DMBX1	Ectoderm	0.14	17.14	0.16	0.07	0.05	2.25
DRD4	Ectoderm	0.08	0.18	0.29	0.28	0.04	1.01
EN1	Ectoderm	0.45	14.79	0.60	0.97	0.21	7.75
LMX1A	Ectoderm	0.00	254.88	0.01	0.05	0.00	125.91
MAP2	Ectoderm	3.90	56.08	3.14	0.23	2.27	21.90
MYO3B	Ectoderm	0.58	0.03	0.67	0.44	0.12	0.19
NOS2	Ectoderm	0.97	2.06	0.50	0.34	0.51	9.09
NR2F1/NR2F2	Ectoderm	0.03	543.33	0.00	0.00	0.00	777.76
NR2F2	Ectoderm	0.04	25.37	0.43	0.17	0.07	16.38
OLFM3	Ectoderm	0.70	94.61	0.31	0.11	0.28	166.29
PAPLN	Ectoderm	0.82	0.02	0.23	0.27	0.63	0.00
PAX3	Ectoderm	0.05	54.90	0.10	0.07	0.07	25.41
PAX6	Ectoderm	0.05	313.84	0.12	0.03	0.06	199.72
POU4F1	Ectoderm	1.75	122.25	0.22	0.60	0.30	180.46
PRKCA	Ectoderm	0.70	2.61	0.55	0.43	0.37	1.33
SDC2	Ectoderm	6.49	13.23	18.15	3.24	8.09	8.50
SOX1	Ectoderm	0.05	0.46	0.07	0.11	0.02	0.92
TRPM8	Ectoderm	0.20	49.15	0.49	0.72	1.03	16.82
WNT1	Ectoderm	0.30	1,121.13	1.23	1.08	0.03	656.31
ZBTB16	Ectoderm	0.20	150.04	0.20	0.25	0.08	224.99

Target Name	Category	undiff Es8	EBs Es8	undiff RFF2	EBs RFF2	undiff Es8 2nd	EBs Es8 2nd
ABCA4	Mesoderm	0.67	79.03	1.10	0.81	0.62	15.88
ALOX15	Mesoderm	1.28	2.30	2.29	0.76	0.58	2.80
BMP10	Mesoderm	1.20	0.10	1.89	0.32	0.92	0.10
CDH5	Mesoderm	2.40	6.17	1.34	0.39	0.77	6.47
CDX2	Mesoderm	0.20	0.09	0.37	0.73	0.05	0.04
COLEC10	Mesoderm	0.85	7.65	0.72	1.66	0.25	12.33
ESM1	Mesoderm	0.64	1,138.26	1.92	2.09	0.67	946.79
FCN3	Mesoderm	1.20	0.01	1.27	0.47	0.32	0.55
FOXF1	Mesoderm	0.21	3.00	0.05	0.39	0.08	3.23
HAND1	Mesoderm	0.03	0.02	0.08	0.03	0.00	0.20
HAND2	Mesoderm	0.02	0.66	0.20	0.25	0.08	2.17
HEY1	Mesoderm	1.36	27.06	0.50	0.35	1.33	53.95
HOPX	Mesoderm	0.28	2.40	2.50	2.62	1.70	2.24
IL6ST	Mesoderm	1.04	12.52	0.89	0.77	0.81	9.77
NKX2-5	Mesoderm	0.45	0.44	1.97	3.29	0.43	1.56
ODAM	Mesoderm	0.96	4.62	1.31	0.17	0.25	3.93
PDGFRA	Mesoderm	0.20	0.81	0.81	0.42	0.59	0.16
PLVAP	Mesoderm	0.53	3.42	1.30	0.54	0.29	1.12
RGS4	Mesoderm	0.14	22.32	0.35	0.21	0.10	11.72
SNAI2	Mesoderm	0.32	19.84	0.50	0.16	0.25	13.91
TBX3	Mesoderm	0.02	6.54	1.03	0.09	0.01	11.16
TM4SF1	Mesoderm	0.92	10.64	0.34	0.14	0.97	14.80
FGF4	Mesendoderm	0.15	0.00	13.69	17.75	0.34	0.00
GDF3	Mesendoderm	4.79	0.00	29.48	38.14	4.50	0.00
NPPB	Mesendoderm	0.02	0.01	2.09	0.16	0.16	0.02
NR5A2	Mesendoderm	181.40	113.46	134.06	70.15	171.18	84.35
PTHLH	Mesendoderm	0.20	26.07	0.74	0.68	2.72	4.67
T	Mesendoderm	0.00	0.00	0.00	0.00	0.00	0.00

Target Name	Category	undiff Es8	EBs Es8	undiff RFF2	EBs RFF2	undiff Es8 2nd	EBs Es8 2nd
AFP	Endoderm	0.01	0.00	0.00	0.00	0.00	0.00
CABP7	Endoderm	0.15	5.89	2.44	8.69	0.18	1.87
CDH20	Endoderm	0.17	50.55	0.29	0.20	0.13	158.58
CLDN1	Endoderm	1.26	105.08	1.59	0.97	0.63	73.47
CPLX2	Endoderm	0.72	17.27	1.63	2.75	0.54	21.62
ELAVL3	Endoderm	0.49	46.43	1.40	2.07	0.37	64.46
EOMES	Endoderm	0.04	1.36	0.03	0.03	0.02	1.75
FOXA1	Endoderm	0.06	0.14	0.12	0.01	0.05	0.10
FOXA2	Endoderm	2.42	0.01	0.01	0.07	2.54	0.01
FOXP2	Endoderm	0.27	12.97	0.05	0.18	0.13	16.40
GATA4	Endoderm	0.01	0.00	0.01	0.03	0.00	0.00
GATA6	Endoderm	0.00	0.00	0.05	0.02	0.01	0.01
HHEX	Endoderm	0.17	0.09	0.16	0.35	0.06	0.01
HMP19	Endoderm	0.66	255.83	0.58	0.55	0.54	121.00
HNF1B	Endoderm	0.17	0.00	0.01	0.01	0.02	0.06
HNF4A	Endoderm	0.01	0.02	0.02	0.01	0.02	0.07
KLF5	Endoderm	0.40	2.88	3.02	1.44	0.76	1.42
LEFTY1	Endoderm	0.01	0.00	0.31	0.26	0.28	0.00
LEFTY2	Endoderm	0.01	0.00	0.15	0.20	0.09	0.00
NODAL	Endoderm	0.04	0.01	0.70	1.10	0.23	0.01
PHOX2B	Endoderm	0.03	3.01	0.06	0.02	0.02	1.17
POU3F3	Endoderm	0.05	18.13	0.07	0.04	0.03	26.48
PRDM1	Endoderm	0.12	0.18	0.10	0.19	0.04	0.36
RXRG	Endoderm	0.19	321.09	0.12	0.17	0.00	316.59
SOX17	Endoderm	0.01	0.01	0.05	0.03	0.01	0.01
SST	Endoderm	0.71	423.43	10.12	3.16	1.27	1,809.87

Target Name	Category	undiff Es8	EBs Es8	undiff RFF2	EBs RFF2	undiff Es8 2nd	EBs Es8 2nd
CXCL5	Self-renewal	7.04	0.01	8.39	4.17	6.59	0.02
DNMT3B	Self-renewal	0.83	0.02	1.18	1.24	0.55	0.03
HESX1	Self-renewal	0.41	0.01	0.24	0.43	0.77	0.01
IDO1	Self-renewal	0.88	0.00	0.67	1.83	0.77	0.00
LCK	Self-renewal	2.29	0.00	1.30	0.70	1.22	0.00
NANOG	Self-renewal	1.02	0.02	2.93	4.06	1.41	0.00
POU5F1	Self-renewal	0.87	0.03	0.87	1.01	0.68	0.01
SOX2	Self-renewal	0.75	0.21	0.73	0.95	1.15	0.28
TRIM22	Self-renewal	3.62	0.20	12.91	4.46	2.44	0.25

Fold change legend

fc > 100	Upregulated
10 < fc <= 100	
2 < fc <= 10	
0.5 <= fc <= 2	Comparable
0.1 <= fc < 0.5	
0.01 <= fc <0.1	
fc < 0.01	Downregulated
omitted	

Figure S1. Gene expression of a subset of genes in ESC in the undifferentiated state and in EBs determined by qRT-PCR.

The expression of 96 genes in KhES-1 categorized as Self-renewal, Ectoderm-. Mesoderm-, Mesendoderm- or Endoderm-lineage before or after EB formation were determined by the qRT-PCR scorecard panel. Notably, genes related to self-renewal were not downregulated nor were those related to differentiation lineagesupregulated in EBs generated from KhES-1 cultured with RFF2. undiff Es8: undifferentiated KhES-1 culture with Es8. EBs Es8: EBs from KhES-1 cultured with Es8. undiff RFF2: undifferentiated KhES-1 cultured with RFF2. EBs RFF2: EBs from KhES-1 cultured with RFF2. undiff Es8 2nd: undifferentiated KhES-1 culture with Es8 again after transferred from RFF2 medium. EBs Es8 2nd: EBs from KhES-1 cultured with Es8 again after transferred from RFF2 medium.

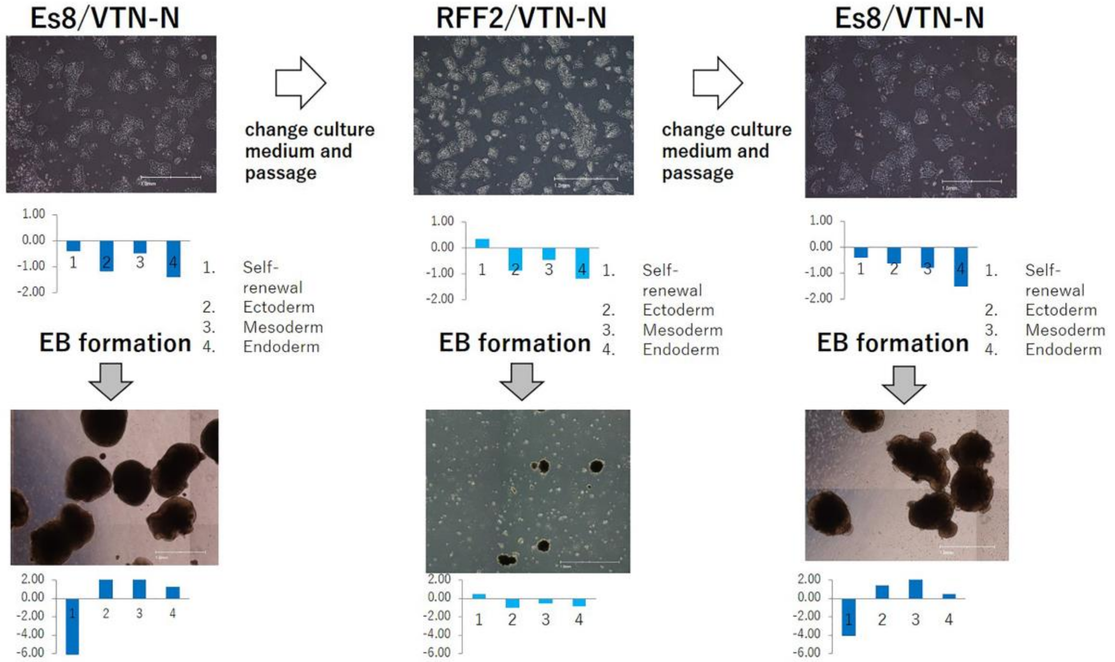

Figure S2. The differentiation potential of iPSCs was altered by culture conditions

The potential for EB formation by iPSCs can be altered reversibly by the culture conditions. iPSCs (PFX#9) in single-cell suspensions were seeded on VNT-N-coated dishes and cultured with Es8 medium (left, upper photograph) for 5 passages. The cells were then collected for EB formation assays (left, lower photograph) or transferred to Repro FF2 medium (RFF2, middle upper photograph). PFX#9 cells were cultured for 5 passages and collected for EB formation assays (middle, lower photo) or transferred to Es8 medium again (right upper photograph). PFX#9 cells were cultured for 5 passages, followed by EB formation assays (right, lower photograph). Photographs of PFX#9 cultures with Es8 or RFF2 (upper panels) at day 1 of culture and EBs on day 14 in EB formation assays (lower panels). Gene expression profiles in cells in the indicated culture conditions were determined using a qRT-PCR scorecard panel and appended below the relevant photograph. Scale bar: 1.0 mm.

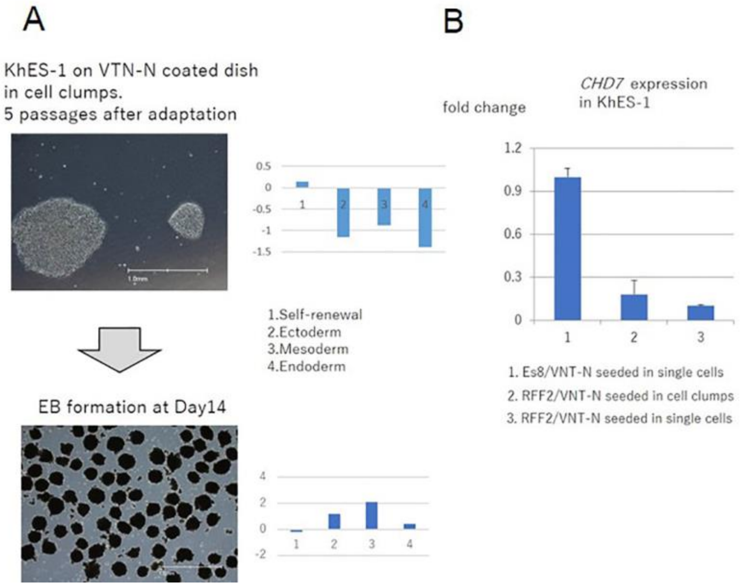

Figure S3. Culture condition alters the differentiation potential of ESC. KhES-1 cells cultured on feeder were transferred to vitronectin-N (VNT-N)-coated dish in cell clumps and cultured for 5 passages for adaptation. The differentiation potential of cells was verified by EB formation assay. A: photos of KhES-1 cells in clumps before EB formation assay and its gene expression profile determined by qRT-PCR scorecard panel are appended in the right (Upper panels). Photos of Day 14-EBs and its genetic profile by scorecard panel (left panels). B. Expression of *CHD7* determined by qRT-PCR in designated culture conditions while maintained in the undifferentiated state (n=3 analytical replicates).

A

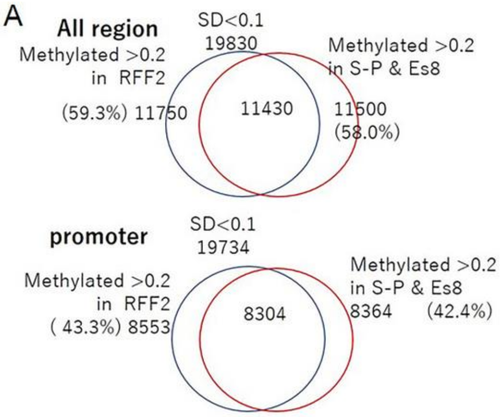

promoter
RFF2 >0.2 and
RFF2/S-P &Es8 ≥ 1.5 ➡ 208 genes

➡ 35 genes

B

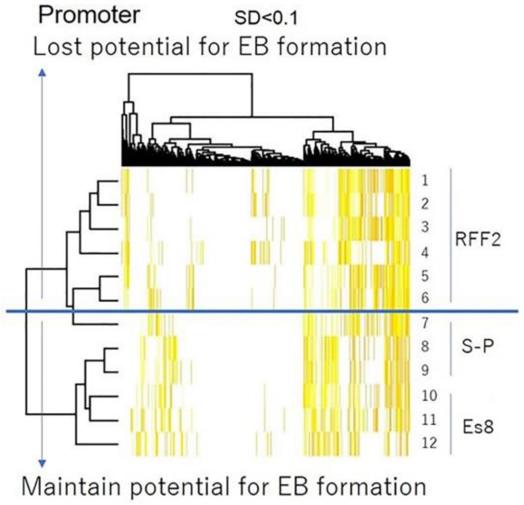

C

self-renewal

GENE_SYMBOL	RFF2	S-P&Es8	Ratio
KLF4	0.02	0.02	1.02
KLF2	0.21	0.19	1.12
TFCP2L1	0.06	0.06	0.97
SOX2	0.02	0.02	0.94
EP300(p300)	0.04	0.04	0.85
ZFP42(REX1)	0.03	0.07	0.43
NANOG	0.36	0.23	1.58
POU5F1	0.26	0.36	0.71
HESX1	0.64	0.59	1.08
LCK	0.73	0.69	1.07
DNMT3B	0.69	0.71	0.97
TRIM22	0.88	0.89	0.98
IDO1	0.92	0.93	0.99

ectoderm

GENE_SYMBOL	RFF2	S-P&Es8	Ratio
COL2A1	0.04	0.04	0.98
DRD4	0.04	0.06	0.78
EN1	0.07	0.05	1.39
LMX1A	0.03	0.03	1.09
NR2F1	0.04	0.03	1.29
NR2F2	0.08	0.06	1.32
OLFM3	0.08	0.07	1.14
PAPLN	0.10	0.11	0.91
PAX3	0.05	0.04	1.45
PAX6	0.03	0.03	1.00
POU4F1	0.03	0.03	1.08
PRKCA	0.03	0.03	0.97
SDC2	0.04	0.03	1.17
SOX1	0.06	0.04	1.47
WNT1	0.11	0.09	1.27
ZBTB16	0.06	0.05	1.22
CDH9	0.35	0.25	1.42
LMX1A	0.84	0.83	1.01
DMBX1	0.82	0.83	0.99
TRPM8	0.89	0.89	1.01
NOS2	0.89	0.90	1.00
MYO3B	0.92	0.93	0.99

mesoderm

GENE_SYMBOL	RFF2	S-P&Es8	Ratio
ALOX15	0.12	0.11	1.10
CDX2	0.03	0.02	1.14
FOXF1	0.04	0.03	1.28
HAND1	0.03	0.03	1.06
HAND2	0.05	0.03	1.56
HEY1	0.01	0.02	0.86
IL6ST	0.04	0.04	1.02
NKX2-5	0.10	0.04	2.75
PDGFRA	0.04	0.03	1.09
SNAI2	0.05	0.03	1.35
TBX3	0.03	0.03	0.98
RGS4	0.33	0.19	1.71
HOPX	0.22	0.22	1.02
ESM1	0.79	0.82	0.97
CDH5	0.85	0.84	1.02
TM4SF1	0.85	0.86	0.99
PLVAP	0.87	0.90	0.98
ABCA4	0.89	0.90	0.98
FCN3	0.90	0.91	0.98
BMP10	0.92	0.92	1.00
COLEC10	0.94	0.94	1.00

mesendoderm

GENE_SYMBOL	RFF2	S-P&Es8	Ratio
FGF4	0.05	0.05	0.89
NPPB	0.14	0.09	1.44
PTHLH	0.03	0.03	1.18
T	0.03	0.03	1.18
GDF3	0.80	0.83	0.97
NR5A2	0.91	0.92	0.99

endoderm

GENE_SYMBOL	RFF2	S-P&Es8	Ratio
CABP7	0.07	0.08	0.85
CLDN1	0.03	0.03	1.01
CPLX2	0.13	0.10	1.29
EOMES	0.07	0.04	1.66
FOXA1	0.03	0.03	1.06
FOXA2	0.04	0.04	0.98
GATA4	0.04	0.03	1.13
GATA6	0.02	0.02	0.91
HHEX	0.03	0.04	0.86
HMP19	0.09	0.07	1.38
HNF1B	0.03	0.03	1.22
KLF5	0.03	0.03	1.04
NODAL	0.06	0.07	0.83
PHOX2B	0.09	0.05	1.75
POU3F3	0.03	0.03	1.04
PRDM1	0.03	0.03	0.94
SOX17	0.04	0.03	1.35
ELAVL3	0.25	0.21	1.16
SST	0.42	0.40	1.05
LEFTY1	0.70	0.74	0.96
FOXP2	0.83	0.78	1.06
LEFTY2	0.84	0.81	1.03
HNF4A	0.91	0.90	1.01
CDH20	0.91	0.93	0.98
AFP	0.92	0.94	0.98

Figure S4. Methylation comparison study of PSCs cultured under various conditions

A. The methylation status of the cells was determined with an Illumina Human Methylation Bead Chip. Average methylation scores from 6 PSC samples cultured with RFF2 (3 iPSC [PFX#9] samples and 3 ESC [KhES-1] samples). Scores were compared with the average of 6 PSC samples in S-P or Es8 medium (1 sample of PFX#9 with S-P; 1 sample of KhES-1 with S-P; 1 sample of H9 with S-P; 1 sample of PFX#9 with Es8; 2 samples of H9 with Es8). The number of gene promoter regions or all regions whose methylation scores exceeded 0.2 by culturing with RFF2 or S-P and Es8 is displayed in a Venn diagram.
B. Clustering of methylation patterns in the promoter region of PSCs cultured with RFF2, S-P, or Es8 medium. Lanes #1–3: iPSCs (PFX#9) cultured with RFF2 medium. Lanes #4–6: ESCs (KhES-1) cultured with RFF2 from 6 independent experiments. Lane #7: PFX#9 cells with S-P. Lane #8: KhES-1 cells with S-P. Lane #9: H9 ESCs with S-P. Lane #10: PFX#9 cells with Es8. Lanes #11, 12: H9 cells with Es8 from 6 independent experiments.
C. Table of methylation status of promoters of representative genes for self-renewal, ectoderm, mesoderm, mesendoderm, and endoderm lineage in PSCs cultured with RFF2, S-P, or Es8 medium. The average methylation status in 6 PSCs (3 iPSCs plus 3 ESCs) cultured with RFF2 or 6 PSCs (1 iPSC sample and 2 ESC samples with S-P and 1 iPSC sample and 2 ESCs with Es8) cultured with S-P or Es8 (S-P & Es8) are shown in the table. All cells were maintained in the undifferentiated state. Methylation status defined as follow: hypomethylated, < 0.2; moderate, 0.2 to < 0.5; hypermethylated, > 0.5. Shown in blue, yellow, and orange columns, respectively.

Target Name	Category	Day 4 Non-transfected control	Day 4 siCHD7	Day 4 mock	Day 5 Non-transfected control	Day 5 siCHD7	Day 5 mock	Day 14 Non-transfected control	Day 14 siCHD7	Day 14 mock
CDH9	Ectoderm	21.84	6.58	24.33	12.24	14.17	15.60	0.76	9.90	0.92
COL2A1	Ectoderm	0.27	0.30	0.43	21.39	1.75	4.02	2.46	16.59	2.15
DMBX1	Ectoderm	59.16	27.86	84.61	98.25	76.12	86.97	17.14	47.83	2.25
DRD4	Ectoderm	1.87	0.94	1.23	0.96	1.50	1.54	0.18	7.08	1.01
EN1	Ectoderm	11.68	14.37	29.77	273.00	10.30	23.02	14.79	156.80	7.75
LMX1A	Ectoderm	15.69	3.63	15.28	27.40	31.49	38.62	254.88	25.25	125.91
MAP2	Ectoderm	5.59	3.46	4.10	12.01	11.69	13.90	56.08	18.50	21.90
MYO3B	Ectoderm	8.04	2.22	8.67	9.81	8.11	11.68	0.03	99.81	0.19
NOS2	Ectoderm	0.16	0.32	0.07	4.86	2.49	10.81	2.06	149.85	9.09
NR2F1/NR2F2	Ectoderm	0.10	0.05	0.08	67.72	3.59	14.40	543.33	91.65	777.76
NR2F2	Ectoderm	15.79	2.66	9.75	9.54	13.92	30.12	25.37	157.80	16.38
OLFM3	Ectoderm	11.37	6.83	23.46	48.67	29.47	36.91	94.61	49.54	166.29
PAPLN	Ectoderm	2.47	1.71	1.47	1.34	1.45	0.97	0.02	1.98	0.00
PAX3	Ectoderm	31.41	2.36	34.57	219.52	152.73	349.60	54.90	211.02	25.41
PAX6	Ectoderm	6.50	0.50	2.63	7.50	15.46	21.43	313.84	307.68	199.72
POU4F1	Ectoderm	3.12	6.97	10.40	22.68	0.48	7.71	122.25	123.41	180.46
PRKCA	Ectoderm	0.54	0.43	0.33	0.87	0.47	0.49	2.61	0.31	1.33
SDC2	Ectoderm	11.85	10.25	14.39	17.14	19.91	18.58	13.23	21.92	8.50
SOX1	Ectoderm	0.03	0.47	0.17	0.57	0.14	0.36	0.46	2.33	0.92
TRPM8	Ectoderm	27.57	3.50	13.04	10.55	8.69	15.14	49.15	25.84	16.82
WNT1	Ectoderm	86.76	15.67	87.34	1,534.88	539.39	689.73	1,121.13	868.42	656.31
ZBTB16	Ectoderm	0.54	2.57	1.62	31.61	8.27	27.43	150.04	183.18	224.99
ABCA4	Mesoderm	5.12	3.45	6.40	8.25	3.44	7.95	79.03	24.07	15.88
ALOX15	Mesoderm	1.34	0.88	0.90	0.82	1.60	0.78	2.30	0.45	2.80
BMP10	Mesoderm	0.21	6.83	8.38	0.58	5.36	0.36	0.10	0.26	0.10
CDH5	Mesoderm	1.60	2.48	0.43	5.15	3.98	3.61	6.17	1.00	6.47
CDX2	Mesoderm	1.10	4.32	4.26	5.02	0.45	0.99	0.09	1.97	0.04
COLEC10	Mesoderm	0.92	0.48	0.93	6.55	8.69	2.96	7.65	1.09	12.33
ESM1	Mesoderm	0.06	3.06	0.36	0.16	0.28	1.14	1,138.26	1.59	946.79
FCN3	Mesoderm	0.52	0.72	0.10	2.17	4.75	1.39	0.01	0.10	0.55
FOXF1	Mesoderm	2.56	8.53	7.71	12.82	2.56	3.22	3.00	0.98	3.23
HAND1	Mesoderm	2.99	2.51	3.30	3.02	1.86	1.74	0.02	0.15	0.20
HAND2	Mesoderm	2.29	1.67	3.03	5.00	0.29	3.75	0.66	3.32	2.17
HEY1	Mesoderm	2.27	2.34	1.78	7.01	3.00	3.56	27.06	4.60	53.95
HOPX	Mesoderm	27.35	11.35	52.63	28.58	23.10	38.80	2.40	5.10	2.24
IL6ST	Mesoderm	0.81	1.22	1.11	0.82	0.73	0.66	12.52	1.18	9.77
NKX2-5	Mesoderm	2.34	3.23	16.27	3.98	5.04	3.20	0.44	0.51	1.56
ODAM	Mesoderm	0.19	0.61	1.18	0.52	0.90	1.77	4.62	2.26	3.93
PDGFRA	Mesoderm	0.97	0.69	0.66	1.18	0.94	0.66	0.81	0.37	0.16
PLVAP	Mesoderm	0.59	0.29	0.11	2.53	0.80	0.66	3.42	1.11	1.12
RGS4	Mesoderm	0.40	0.29	0.19	0.98	0.52	0.62	22.32	0.40	11.72
SNAI2	Mesoderm	0.66	0.68	1.54	34.49	5.58	13.15	19.84	11.63	13.91
TBX3	Mesoderm	0.27	0.62	0.71	1.77	0.28	0.57	6.54	0.51	11.16
TM4SF1	Mesoderm	1.96	1.15	1.89	0.88	1.51	1.25	10.64	0.22	14.80
FGF4	Mesendoderm	2.32	1.64	1.62	2.04	1.99	1.15	0.00	0.18	0.00
GDF3	Mesendoderm	1.55	3.71	1.28	5.33	1.45	1.21	0.00	0.02	0.00
NPPB	Mesendoderm	0.08	0.53	0.18	0.09	0.11	0.05	0.01	0.00	0.02
NR5A2	Mesendoderm	70.03	69.50	69.59	120.59	49.58	64.03	113.46	7.25	84.35
PTHLH	Mesendoderm	6.99	54.08	20.75	9.29	12.33	10.77	26.07	0.19	4.67
T	Mesendoderm	0.00	0.00	0.00	0.05	0.01	0.01	0.00	0.00	0.00

Target Name	Category	Day 4 Non-transfected control	Day 4 siCHD7	Day 4 mock	Day 5 Non-transfected control	Day 5 siCHD7	Day 5 mock	Day 14 Non-transfected control	Day 14 siCHD7	Day 14 mock
AFP	Endoderm	0.00	0.06	0.02	0.01	0.02	0.01	0.00	0.00	0.00
CABP7	Endoderm	14.04	10.74	11.74	8.53	6.08	5.30	5.89	0.65	1.87
CDH20	Endoderm	4.53	0.83	2.71	8.97	2.30	4.72	50.55	8.42	158.58
CLDN1	Endoderm	0.55	1.91	1.20	1.40	0.51	1.02	105.08	3.26	73.47
CPLX2	Endoderm	2.31	2.48	1.02	1.66	1.01	1.23	17.27	0.49	21.62
ELAVL3	Endoderm	0.92	0.79	0.74	1.15	0.48	0.62	46.43	0.83	64.46
EOMES	Endoderm	0.08	0.17	0.22	2.35	0.11	0.30	1.36	0.01	1.75
FOXA1	Endoderm	0.74	0.52	1.95	1.41	0.73	1.11	0.14	2.03	0.10
FOXA2	Endoderm	0.08	0.20	0.16	0.45	0.08	0.11	0.01	0.02	0.01
FOXP2	Endoderm	1.39	1.42	3.52	3.15	0.66	0.67	12.97	1.37	16.40
GATA4	Endoderm	0.04	0.09	0.09	1.05	0.17	0.17	0.00	0.00	0.00
GATA6	Endoderm	0.01	0.03	0.02	0.15	0.01	0.02	0.00	0.03	0.01
HHEX	Endoderm	1.04	0.51	0.72	0.23	0.43	0.25	0.09	0.04	0.01
HMP19	Endoderm	0.20	3.07	3.76	2.83	0.18	0.22	255.83	2.28	121.00
HNF1B	Endoderm	0.07	0.01	0.13	0.15	0.01	0.02	0.00	0.01	0.06
HNF4A	Endoderm	0.06	0.01	0.23	0.25	0.07	0.12	0.02	0.11	0.07
KLF5	Endoderm	0.91	2.36	1.79	1.60	1.40	1.81	2.88	0.44	1.42
LEFTY1	Endoderm	0.01	0.06	0.01	0.06	0.01	0.02	0.00	0.01	0.00
LEFTY2	Endoderm	0.02	0.11	0.04	0.23	0.05	0.07	0.00	0.00	0.00
NODAL	Endoderm	0.06	0.45	0.16	0.60	0.16	0.25	0.01	0.01	0.01
PHOX2B	Endoderm	0.10	0.33	0.64	0.28	0.49	0.17	3.01	0.02	1.17
POU3F3	Endoderm	0.19	0.28	0.73	1.75	0.19	0.47	18.13	1.28	26.48
PRDM1	Endoderm	1.48	1.82	2.65	1.34	2.10	1.72	0.18	0.45	0.36
RXRG	Endoderm	1.68	1.29	5.33	7.87	3.31	3.96	321.09	3.87	316.59
SOX17	Endoderm	0.03	0.11	0.20	1.15	0.07	0.03	0.01	0.04	0.01
SST	Endoderm	1.40	0.93	5.57	33.25	0.50	5.64	423.43	5.49	1,809.87
CXCL5	Self-renewal	1.72	2.16	1.19	0.79	0.62	0.55	0.01	0.03	0.02
DNMT3B	Self-renewal	0.67	0.58	0.58	0.43	0.44	0.32	0.02	0.08	0.03
HESX1	Self-renewal	1.09	0.27	0.67	0.31	0.76	0.57	0.01	0.33	0.01
IDO1	Self-renewal	0.49	0.42	0.24	0.14	0.17	0.10	0.00	0.00	0.00
LCK	Self-renewal	0.27	0.22	0.14	0.15	0.12	0.11	0.00	0.01	0.00
NANOG	Self-renewal	0.38	0.79	0.49	0.57	0.37	0.22	0.02	0.01	0.00
POU5F1	Self-renewal	0.45	0.68	0.36	0.26	0.16	0.11	0.03	0.01	0.01
SOX2	Self-renewal	1.55	1.08	1.75	0.85	1.48	1.16	0.21	1.89	0.28
TRIM22	Self-renewal	4.42	7.18	5.03	2.96	3.81	2.94	0.20	0.41	0.25

Fold change legend

fc > 100	Upregulated
10 < fc <= 100	
2 < fc <= 10	
0.5 <= fc <= 2	Comparable
0.1 <= fc < 0.5	
0.01 <= fc <0.1	
fc < 0.01	Downregulated
omitted	

Figure S5. Table for 96 genes expression of *mock*- or si*CHD7*-transfected KhES-1 in EB formation assay day 4, 5 or 14 determined by qRT-PCR scorecard panel. This is a raw data before normalization for bar graphs in Figure 2D.

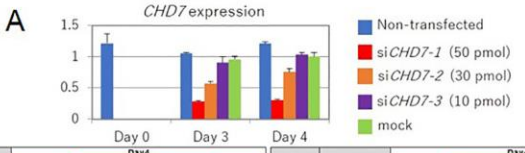

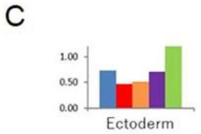

B

Target Name	Category	Day4 Non-transfected control	Day4 siCHD7-1	Day4 siCHD7-2	Day4 siCHD7-3	Day4 mock
CDH9	Ectoderm	60.65	9.00	8.86	39.74	138.43
COL2A1	Ectoderm	0.38	0.40	0.32	0.28	0.57
DMBX1	Ectoderm	21.92	19.21	21.25	30.84	56.69
DRD4	Ectoderm	0.43	0.20	0.26	0.34	0.86
EN1	Ectoderm	0.89	5.17	4.73	3.34	3.11
LMX1A	Ectoderm	29.83	21.22	17.53	30.47	65.59
MAP2	Ectoderm	11.38	5.86	7.45	9.94	20.83
MYO3B	Ectoderm	2.15	0.88	1.14	1.95	3.43
NOS2	Ectoderm	0.53	0.33	0.30	0.32	0.88
NR2F1/NR2F2	Ectoderm	0.58	0.44	0.19	0.31	1.21
NR2F2	Ectoderm	13.14	5.58	2.37	7.27	13.23
OLFM3	Ectoderm	3.84	2.88	3.61	5.16	10.56
PAPLN	Ectoderm	0.53	1.02	1.03	0.97	1.83
PAX3	Ectoderm	16.50	3.07	10.28	8.67	23.57
PAX6	Ectoderm	44.23	12.49	14.64	32.92	84.13
POU4F1	Ectoderm	0.48	1.22	1.09	0.74	1.97
PRKCA	Ectoderm	0.25	0.34	0.65	0.38	0.57
SDC2	Ectoderm	9.55	9.83	8.54	9.17	23.48
SOX1	Ectoderm	0.47	0.55	0.33	0.43	1.08
TRPM8	Ectoderm	16.90	10.31	12.00	16.97	42.42
WNT1	Ectoderm	60.73	47.32	45.49	55.05	131.95
ZBTB16	Ectoderm	1.78	0.46	0.35	1.03	2.20

Fold change legend	
fc > 100	Upregulated
10 < fc <= 100	
2 < fc <= 10	
0.5 <= fc <= 2	Comparable
0.1 <= fc < 0.5	
0.01 <= fc <0.1	
fc < 0.01	Downregulated
omitted	

Target Name	Category	Day4 Non-transfected control	Day4 siCHD7-1	Day4 siCHD7-2	Day4 siCHD7-3	Day4 mock
ABCA4	Mesoderm	1.50	0.74	0.45	0.69	1.92
ALOX15	Mesoderm	0.18	0.20	0.25	0.21	0.51
BMP10	Mesoderm	0.35	0.05	0.04	0.04	0.11
CDH5	Mesoderm	1.70	1.56	1.52	2.07	4.50
CDX2	Mesoderm	0.09	0.06	0.01	0.12	0.32
COLEC10	Mesoderm	0.38	0.30	0.31	0.38	0.93
ESM1	Mesoderm	0.28	0.01	0.47	0.16	0.52
FCN3	Mesoderm	0.24	0.02	0.08	0.15	0.29
FOXF1	Mesoderm	1.32	0.56	0.46	1.04	2.00
HAND1	Mesoderm	0.21	0.14	0.12	0.19	0.50
HAND2	Mesoderm	1.41	1.98	0.91	1.71	3.16
HEY1	Mesoderm	1.08	0.87	0.84	1.22	2.97
HOPX	Mesoderm	3.12	1.41	1.45	3.09	6.80
IL6ST	Mesoderm	0.44	0.50	0.40	0.41	0.96
NKX2-5	Mesoderm	1.36	0.98	0.57	1.16	3.91
ODAM	Mesoderm	0.04	0.04	0.04	0.34	0.10
PDGFRA	Mesoderm	0.31	0.18	0.28	0.34	0.64
PLVAP	Mesoderm	0.52	0.36	0.50	0.54	1.16
RGS4	Mesoderm	0.53	0.33	0.35	0.35	0.61
SNAI2	Mesoderm	0.23	0.15	0.23	0.20	0.53
TBX3	Mesoderm	0.36	0.10	0.10	0.25	0.57
TM4SF1	Mesoderm	0.88	0.36	0.98	0.74	2.25
FGF4	Mesendoderm	0.45	0.56	0.35	0.39	0.63
GDF3	Mesendoderm	0.07	0.41	0.40	0.18	0.40
NPPB	Mesendoderm	0.00	0.00	0.00	0.00	0.00
NR5A2	Mesendoderm	3.95	7.34	13.55	5.85	11.66
PTHLH	Mesendoderm	0.40	0.16	0.98	0.35	1.15
T	Mesendoderm	0.00	0.00	0.00	0.00	0.00

Target Name	Category	Day4 Non-transfected control	Day4 siCHD7-1	Day4 siCHD7-2	Day4 siCHD7-3	Day4 mock
AFP	Endoderm	0.00	0.00	0.00	0.00	0.00
CABP7	Endoderm	0.36	1.50	1.74	1.92	1.50
CDH20	Endoderm	1.62	1.70	1.61	1.89	3.73
CLDN1	Endoderm	0.19	0.22	0.25	0.21	0.51
CPLX2	Endoderm	0.21	0.56	0.36	0.61	0.76
ELAVL3	Endoderm	0.09	0.06	0.13	0.14	0.19
EOMES	Endoderm	0.03	0.03	0.03	0.03	0.10
FOXA1	Endoderm	0.09	0.06	0.05	0.04	0.20
FOXA2	Endoderm	0.01	0.01	0.01	0.01	0.02
FOXP2	Endoderm	0.20	0.05	0.16	0.19	0.50
GATA4	Endoderm	0.01	0.00	0.00	0.01	0.01
GATA6	Endoderm	0.01	0.02	0.01	0.02	0.03
HHEX	Endoderm	1.47	1.17	1.31	1.70	3.70
HMP19	Endoderm	0.42	0.39	0.63	0.50	2.39
HNF1B	Endoderm	0.00	0.01	0.00	0.00	0.00
HNF4A	Endoderm	0.05	0.01	0.01	0.04	0.08
KLF5	Endoderm	0.25	0.20	0.22	0.25	0.57
LEFTY1	Endoderm	0.00	0.00	0.00	0.00	0.00
LEFTY2	Endoderm	0.00	0.00	0.00	0.01	0.01
NODAL	Endoderm	0.00	0.00	0.00	0.00	0.00
PHOX2B	Endoderm	0.02	0.02	0.02	0.02	0.05
POU3F3	Endoderm	0.05	0.06	0.06	0.07	0.16
PRDM1	Endoderm	0.48	0.11	0.15	0.43	1.27
RXRG	Endoderm	1.30	4.69	1.04	1.11	2.99
SOX17	Endoderm	0.01	0.02	0.02	0.01	0.02
SST	Endoderm	0.49	0.18	0.15	0.33	0.41
CXCL5	Self-renewal	0.13	0.10	0.18	0.18	0.46
DNMT3B	Self-renewal	0.18	0.18	0.28	0.25	0.44
HESX1	Self-renewal	3.34	4.18	2.77	2.98	6.78
IDO1	Self-renewal	0.08	0.14	0.38	0.14	0.33
LCK	Self-renewal	0.07	0.06	0.11	0.10	0.30
NANOG	Self-renewal	0.01	0.01	0.02	0.01	0.03
POU5F1	Self-renewal	0.01	0.02	0.05	0.02	0.05
SOX2	Self-renewal	3.01	2.12	1.97	2.73	6.64
TRIM22	Self-renewal	1.47	2.39	1.86	2.43	5.18

Figure S6. Differentiation potential of KhES-1 correlates to the expression level of *CHD7*. *CHD7* in KhES-1 was downregulated by introducing either 10, 30 or 50 pmol of si*CHD7* into cells (A) subsequently, differentiation potential was determined by qRT-PCR of 96 genes with a scorecard panel (B). Ectoderm differentiation potential after normalized was as bar graph (C).

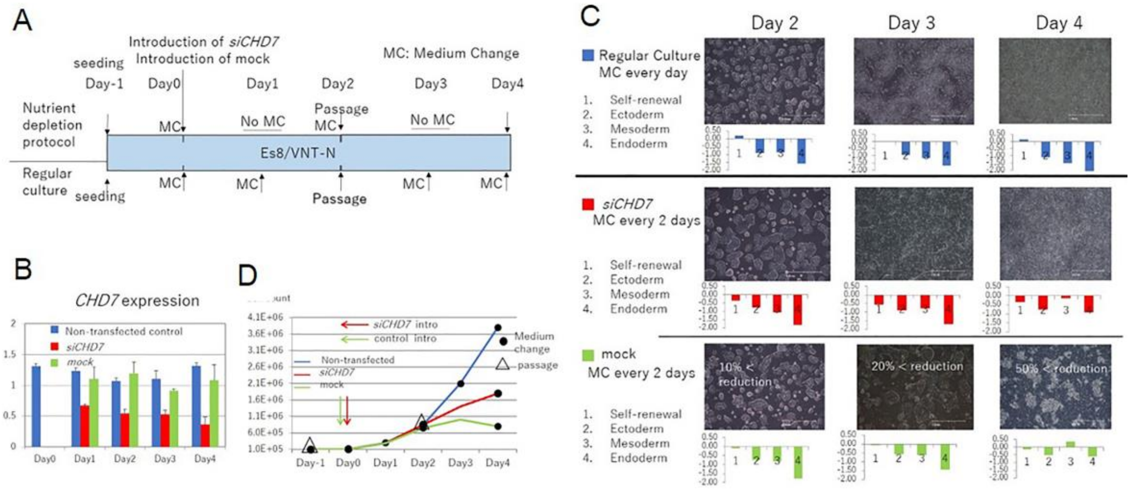

Figure S7. Downregulation of *CHD7* supported the survival of ESCs cultured with nutrient-depleted media.

A. Protocol for induction of differentiation by nutrient-depleted Es8 medium and transfection with *siCHD7*. *siCHD7* or control siRNA (mock) was transfected into cells after changing the medium (Es8) on day 0. The medium was then changed every 2 days (days 2 and 4). Cells were harvested 42 h (day 2), 72 h, and 96 h after transfection with siRNA for cell counting and determination of gene expression by qRT-PCR. ESCs were normally cultured in Es8 medium with daily medium changes and passaged every 3 days to maintain an undifferentiated state.

B. *CHD7* gene expression was determined by qRT-PCR in KhES-1 cells transfected with *siCHD7* or control siRNA (mock) or in non-transfected cells on days 0, 1, 2, 3 and 4. *CHD7* gene expression was standardized according to the average *CHD7* expression in KhES-1 cells cultured with Es8 medium measured independently 3 times.

C. Photomicrographs of non-transfected regular cultures of KhES-1 cells (upper panels, daily medium changes), *siCHD7*-transfected KhES-1 cells (middle panels, medium changes every 2 days), and control siRNA-transfected (mock) KhES-1 cells (lower panels, medium changes every 2 days) on days 2, 3, and 4 are shown. Gene expression profiles of KhES-1 cells, as determined by qRT-PCR scorecard panel, are shown below the corresponding photograph. Scale bar: 1 mm.

D. Numbers of non-transfected KhES-1 cells (blue line), *siCHD7*-transfected cells (red line), or mock-transfected cells (green line) on the designated cultured day are plotted as a linear graph. Representative data sets from 3 independent experiments are shown.

Target Name	Category	Day1 Non-transfected control	Day1 mCHD7	Day1 mock	Day2 Non-transfected control	Day2 mCHD7	Day2 mock	Day3 Non-transfected control	Day3 mCHD7	Day3 mock
CDH9	Ectoderm	0.01	0.36	0.02	0.04	0.08	0.01	0.01	0.05	0.03
COL2A1	Ectoderm	0.21	1.75	0.19	0.77	3.09	0.43	0.35	1.03	0.33
DMBX1	Ectoderm	0.42	0.40	0.34	0.16	0.51	0.22	0.13	0.38	0.10
DRD4	Ectoderm	0.22	0.50	0.24	0.32	0.46	0.22	0.33	0.56	0.33
EN1	Ectoderm	0.65	69.19	1.23	0.87	8.41	2.96	0.44	2.43	0.36
LMX1A	Ectoderm	0.00	2.64	0.08	0.01	2.46	0.29	0.00	0.48	0.02
MAP2	Ectoderm	7.24	10.18	11.63	14.79	14.96	16.34	2.66	5.35	3.86
MYO3B	Ectoderm	0.43	0.35	0.29	0.76	0.32	2.36	0.54	0.65	0.81
NOS2	Ectoderm	0.82	1.31	0.75	0.70	1.68	1.08	0.77	0.68	0.90
NR2F1/NR2F2	Ectoderm	0.00	21.43	0.03	0.01	28.47	0.31	0.01	7.33	0.06
NR2F2	Ectoderm	0.30	3.44	0.23	0.63	1.56	1.10	0.32	1.38	0.41
OLFM3	Ectoderm	0.68	2.00	0.57	0.39	1.23	0.44	0.27	0.77	0.46
PAPLN	Ectoderm	0.19	0.41	0.21	0.44	0.43	0.34	0.36	0.35	0.39
PAX3	Ectoderm	0.58	59.94	0.33	1.94	12.68	0.50	0.07	5.65	0.10
PAX6	Ectoderm	0.13	16.25	0.18	0.11	10.65	0.13	0.04	2.26	0.11
POU4F1	Ectoderm	0.40	42.21	0.04	0.04	33.30	0.10	0.49	7.77	0.32
PRKCA	Ectoderm	0.77	0.57	0.75	0.79	0.84	0.72	0.65	0.78	0.72
SDC2	Ectoderm	29.34	20.10	19.66	26.51	18.81	18.98	17.11	15.36	16.78
SOX1	Ectoderm	0.05	0.22	0.07	0.11	0.19	0.02	0.19	0.14	0.10
TRPM8	Ectoderm	0.57	0.37	0.67	0.28	0.63	0.78	1.59	1.53	0.93
WNT1	Ectoderm	1.73	3.56	4.36	1.45	4.66	1.49	2.25	10.01	1.82
ZBTB16	Ectoderm	0.24	0.58	0.27	0.27	0.74	0.36	0.47	0.62	0.27
ABCA4	Mesoderm	0.59	0.87	0.75	0.99	2.17	0.92	1.17	2.41	1.80
ALOX15	Mesoderm	2.62	2.67	2.32	3.03	2.18	2.74	3.13	2.35	3.47
BMP10	Mesoderm	1.29	3.39	1.24	3.85	3.14	2.46	0.32	0.06	1.29
CDH5	Mesoderm	6.21	3.01	4.44	4.59	7.23	11.32	1.09	8.08	3.76
CDX2	Mesoderm	0.15	8.22	0.16	0.05	10.09	0.32	0.27	6.01	0.66
COLEC10	Mesoderm	0.49	0.72	1.09	1.65	2.39	2.72	1.95	1.41	2.17
ESM1	Mesoderm	1.83	1.74	1.58	0.03	0.51	0.56	2.82	3.67	2.68
FCN3	Mesoderm	0.69	0.66	0.58	1.46	0.95	1.02	1.76	0.47	1.17
FOXF1	Mesoderm	0.30	13.58	0.51	0.69	17.82	1.58	0.18	6.37	0.92
HAND1	Mesoderm	0.18	1.57	0.26	0.14	1.66	0.70	0.10	5.67	0.89
HAND2	Mesoderm	0.37	273.66	0.63	0.02	317.00	1.04	0.24	60.26	0.72
HEY1	Mesoderm	0.74	1.33	0.65	0.59	1.01	0.77	0.44	0.72	0.51
HOPX	Mesoderm	1.90	15.67	0.40	2.09	2.38	0.50	0.28	1.46	0.44
IL6ST	Mesoderm	1.15	1.85	1.51	1.05	3.57	2.05	0.81	2.49	1.36
NKX2-5	Mesoderm	2.60	33.32	2.56	2.65	25.85	3.16	2.11	6.08	2.34
ODAM	Mesoderm	0.96	15.69	1.71	1.30	4.54	1.48	0.53	1.87	1.40
PDGFRA	Mesoderm	1.25	0.57	0.84	0.70	0.36	0.84	0.51	0.73	0.81
PLVAP	Mesoderm	1.23	1.32	0.78	1.70	1.85	1.32	2.12	1.19	1.53
RGS4	Mesoderm	0.77	1.53	0.32	0.30	3.24	0.37	1.27	2.02	1.48
SNAI2	Mesoderm	0.60	0.73	0.72	0.39	0.75	0.59	0.32	0.97	0.46
TBX3	Mesoderm	1.42	3.02	1.83	0.59	3.39	1.24	0.85	2.82	1.92
TM4SF1	Mesoderm	0.38	0.46	0.22	0.37	0.46	0.39	0.39	0.99	0.42
FGF4	Mesendoderm	10.49	5.04	5.56	12.41	5.41	11.18	12.77	18.31	16.21
GDF3	Mesendoderm	23.80	9.35	15.71	26.97	12.60	22.59	42.30	26.71	37.12
NPPB	Mesendoderm	3.15	12.79	7.14	2.16	16.30	4.78	0.79	7.36	2.09
NR5A2	Mesendoderm	128.86	259.54	122.08	161.63	313.07	161.89	184.43	174.98	179.10
PTHLH	Mesendoderm	1.02	45.09	1.24	0.52	32.69	1.36	0.28	9.52	0.73
T	Mesendoderm	0.00	0.01	0.00	0.00	0.01	0.00	0.00	0.02	0.00

Target Name	Category	Day1 Non-transfected control	Day1 mCHD7	Day1 mock	Day2 Non-transfected control	Day2 mCHD7	Day2 mock	Day3 Non-transfected control	Day3 mCHD7	Day3 mock
AFP	Endoderm	0.04	0.32	0.03	0.00	0.10	0.01	0.00	0.06	0.00
CABP7	Endoderm	2.02	5.18	2.05	2.70	3.55	2.48	3.66	8.92	5.14
CDH20	Endoderm	0.75	3.28	0.39	0.36	3.05	1.01	0.34	1.26	0.45
CLDN1	Endoderm	1.84	3.95	2.30	2.79	4.35	2.95	1.78	2.40	2.16
CPLX2	Endoderm	2.34	4.33	2.17	1.64	2.91	2.27	2.45	3.19	2.99
ELAVL3	Endoderm	0.81	3.33	0.88	3.67	2.33	2.07	2.45	0.85	1.70
EOMES	Endoderm	0.02	13.59	0.07	0.05	5.22	0.05	0.03	1.94	0.03
FOXA1	Endoderm	0.10	21.37	0.07	0.07	14.07	0.19	0.02	4.44	0.09
FOXA2	Endoderm	0.01	0.11	0.03	0.04	0.27	0.05	0.04	0.29	0.05
FOXP2	Endoderm	0.24	3.96	0.12	0.22	7.37	0.32	0.12	2.35	0.16
GATA4	Endoderm	0.02	4.26	0.09	0.03	3.65	0.10	0.03	1.21	0.06
GATA6	Endoderm	0.10	2.67	0.27	0.08	2.40	0.48	0.03	1.28	0.25
HHEX	Endoderm	0.10	4.36	0.11	0.16	2.62	0.12	0.13	1.01	0.21
HMP19	Endoderm	0.44	0.52	0.52	0.80	1.31	0.72	1.33	0.75	0.76
HNF1B	Endoderm	0.02	0.36	0.02	0.03	0.32	0.05	0.01	0.22	0.05
HNF4A	Endoderm	0.03	0.87	0.12	0.02	0.89	0.26	0.02	0.31	0.14
KLF5	Endoderm	4.02	19.30	6.91	4.14	11.28	5.08	1.97	6.25	2.96
LEFTY1	Endoderm	0.44	0.31	0.36	0.35	0.30	0.43	0.26	0.23	0.28
LEFTY2	Endoderm	0.26	0.14	0.23	0.11	0.23	0.16	0.05	0.14	0.09
NODAL	Endoderm	1.11	0.95	1.06	1.19	1.40	1.57	0.71	1.15	0.70
PHOX2B	Endoderm	0.03	2.55	0.03	0.03	1.73	0.03	0.03	0.40	0.04
POU3F3	Endoderm	0.05	3.46	0.08	0.05	1.53	0.06	0.08	0.44	0.07
PRDM1	Endoderm	0.21	1.74	0.17	0.31	3.66	0.49	0.34	1.70	0.44
RXRG	Endoderm	0.05	2.07	0.98	0.01	6.17	0.86	0.05	2.02	0.35
SOX17	Endoderm	0.07	54.39	0.07	0.12	4.12	0.11	0.01	1.76	0.25
SST	Endoderm	12.92	12.19	10.67	10.84	17.65	14.97	19.21	23.81	16.72
CXCL5	Self-renewal	12.60	7.18	10.05	8.51	5.12	6.78	6.31	4.87	6.68
DNMT3B	Self-renewal	1.23	0.82	1.01	1.17	0.84	1.46	1.80	1.39	1.64
HESX1	Self-renewal	0.41	0.24	0.28	0.26	0.21	0.21	0.37	0.32	0.39
IDO1	Self-renewal	0.55	0.38	0.31	0.76	0.41	0.48	0.90	0.68	0.83
LCK	Self-renewal	1.28	1.07	0.99	1.70	0.92	1.54	2.01	1.10	1.76
NANOG	Self-renewal	3.09	2.19	2.94	4.67	2.42	3.17	3.92	2.62	3.24
POU5F1	Self-renewal	0.98	0.55	0.64	1.80	1.35	1.30	1.63	0.77	1.30
SOX2	Self-renewal	0.76	0.65	0.56	0.71	0.71	0.61	0.74	0.78	0.67
TRIM22	Self-renewal	21.76	34.22	19.35	28.64	17.95	22.26	17.20	14.86	21.28

Fold change legend

fc > 100	Upregulated
10 < fc <= 100	
2 < fc <= 10	
0.5 <= fc <= 2	Comparable
0.1 <= fc < 0.5	
0.01 <= fc < 0.1	
fc < 0.01	Downregulated
omitted	

Figure S8. Gene expression data from ESC after introduction of *mCHD7* determined by the qRT-PCR.

mRNA encoding *CHD7* isoform 2 (mCHD7) or *mock* RNA (mock) was transfected into KhES-1 cells cultured with RFF2 medium, and the expression levels of 96 genes in KhES-1 at 1, 2, and 3 days after transfection were determined by the qRT-PCR scorecard panel. Notably, three germ layer differentiation was observed simultaneously with upregulation of *CHD7* isoform 2 mRNA.

		Day 0	Day 3				
Target Name	Category	Non-transfected control	Non-transfected control	CHD7 DN1	CHD7 DN2	CHD7 DN1+DN2	mock
CDH9	Ectoderm	0.09	33.24	36.89	21.58	25.70	33.72
COL2A1	Ectoderm	0.21	0.28	0.26	0.25	0.43	0.63
DMBX1	Ectoderm	0.46	12.58	10.76	10.26	7.39	14.48
DRD4	Ectoderm	0.00	0.29	0.18	0.16	0.23	0.30
EN1	Ectoderm	0.43	2.16	0.45	0.62	0.40	1.47
LMX1A	Ectoderm	0.20	11.45	7.56	4.39	4.52	7.21
MAP2	Ectoderm	1.33	9.79	8.59	7.47	8.56	10.07
MYO3B	Ectoderm	0.08	2.04	0.92	0.48	0.45	2.19
NOS2	Ectoderm	0.77	0.30	0.30	0.30	0.31	0.34
NR2F1/NR2F2	Ectoderm	0.01	0.04	0.01	0.01	0.04	0.16
NR2F2	Ectoderm	0.01	0.44	0.31	0.22	0.21	0.38
OLFM3	Ectoderm	0.97	1.86	1.70	1.01	1.40	1.68
PAPLN	Ectoderm	0.12	1.47	1.41	1.18	1.00	1.80
PAX3	Ectoderm	0.04	1.50	0.13	0.06	0.05	1.45
PAX6	Ectoderm	0.04	2.89	1.36	0.33	0.38	1.92
POU4F1	Ectoderm	0.54	1.50	0.81	0.56	1.31	1.29
PRKCA	Ectoderm	0.63	0.65	0.77	0.81	1.05	0.67
SDC2	Ectoderm	3.19	16.59	13.11	9.03	8.23	15.14
SOX1	Ectoderm	0.03	0.64	0.43	0.48	0.63	0.58
TRPM8	Ectoderm	0.38	3.21	2.00	0.55	0.61	2.10
WNT1	Ectoderm	0.05	12.66	4.94	2.17	1.60	9.55
ZBTB16	Ectoderm	0.11	0.16	0.11	0.10	0.08	0.09

		Day 0	Day 3				
Target Name	Category	Non-transfected control	Non-transfected control	CHD7 DN1	CHD7 DN2	CHD7 DN1+DN2	mock
ABCA4	Mesoderm	0.37	0.31	0.10	0.41	0.10	0.74
ALOX15	Mesoderm	1.04	0.30	0.33	0.37	0.47	0.30
BMP10	Mesoderm	0.70	0.71	0.78	0.08	0.06	0.06
CDH5	Mesoderm	3.87	1.20	1.08	0.94	0.79	1.96
CDX2	Mesoderm	0.02	0.25	0.04	0.14	0.00	0.15
COLEC10	Mesoderm	0.26	1.09	0.56	0.69	0.49	2.24
ESM1	Mesoderm	0.08	0.02	0.02	0.02	0.02	0.48
FCN3	Mesoderm	0.50	0.17	0.12	0.15	0.29	0.35
FOXF1	Mesoderm	0.25	0.53	0.58	0.68	0.68	0.75
HAND1	Mesoderm	0.03	0.34	0.19	0.27	0.25	0.37
HAND2	Mesoderm	0.17	0.02	0.12	0.28	0.66	0.46
HEY1	Mesoderm	1.54	1.11	1.00	1.18	1.07	2.12
HOPX	Mesoderm	0.06	2.00	2.09	3.88	4.35	2.82
IL6ST	Mesoderm	0.91	0.50	0.49	0.34	0.34	0.40
NKX2-5	Mesoderm	0.07	0.59	0.32	0.13	0.29	0.33
ODAM	Mesoderm	0.52	0.05	0.06	0.07	3,761.99	0.06
PDGFRA	Mesoderm	0.35	0.78	0.68	0.35	0.41	0.80
PLVAP	Mesoderm	0.66	0.65	0.67	0.70	0.63	0.75
RGS4	Mesoderm	0.13	0.14	0.12	0.27	1.14	0.12
SNAI2	Mesoderm	0.30	0.03	0.06	0.08	0.07	0.05
TBX3	Mesoderm	0.01	0.17	0.04	0.07	0.05	0.12
TM4SF1	Mesoderm	0.89	4.27	4.43	2.56	2.79	4.25
FGF4	Mesendoderm	0.14	0.97	0.23	0.18	0.23	0.83
GDF3	Mesendoderm	3.63	0.51	0.71	0.64	0.60	0.47
NPPB	Mesendoderm	0.01	0.00	0.01	0.01	0.02	0.01
NR5A2	Mesendoderm	83.62	6.73	10.20	12.91	16.46	8.78
PTHLH	Mesendoderm	0.41	1.63	2.46	2.17	3.97	1.64
T	Mesendoderm	0.00	0.00	0.00	0.00	0.00	0.00

		Day 0	Day 3				
Target Name	Category	Non-transfected control	Non-transfected control	CHD7 DN1	CHD7 DN2	CHD7 DN1+DN2	mock
AFP	Endoderm	0.00	0.00	0.00	0.07	0.00	0.00
CABP7	Endoderm	0.06	1.50	1.34	0.93	1.00	1.68
CDH20	Endoderm	0.41	0.79	0.70	0.67	0.78	0.51
CLDN1	Endoderm	0.73	0.49	0.63	0.49	0.58	0.56
CPLX2	Endoderm	0.73	0.92	0.56	1.86	1.85	0.70
ELAVL3	Endoderm	0.24	0.30	0.28	0.19	0.28	0.39
EOMES	Endoderm	0.04	0.08	0.06	0.05	0.07	0.07
FOXA1	Endoderm	0.04	0.04	0.04	0.07	0.05	0.05
FOXA2	Endoderm	1.95	0.01	0.02	0.04	0.05	0.02
FOXP2	Endoderm	0.28	0.11	0.29	0.07	0.10	0.21
GATA4	Endoderm	0.00	0.00	0.00	0.01	0.01	0.01
GATA6	Endoderm	0.00	0.01	0.01	0.01	0.01	0.01
HHEX	Endoderm	0.03	1.13	0.90	0.53	0.56	1.04
HMP19	Endoderm	1.03	0.45	0.39	0.42	0.46	0.26
HNF1B	Endoderm	0.04	0.00	0.00	0.02	0.01	0.00
HNF4A	Endoderm	0.01	0.04	0.01	0.03	0.00	0.03
KLF5	Endoderm	0.22	0.81	0.43	0.34	0.36	0.67
LEFTY1	Endoderm	0.01	0.00	0.00	0.00	0.00	0.02
LEFTY2	Endoderm	0.01	0.01	0.00	0.01	0.01	0.00
NODAL	Endoderm	0.18	0.00	0.01	0.00	0.00	0.00
PHOX2B	Endoderm	0.03	0.03	0.05	0.04	0.03	0.03
POU3F3	Endoderm	0.03	0.06	0.05	0.07	0.08	0.06
PRDM1	Endoderm	0.04	1.38	0.72	1.01	0.62	1.62
RXRG	Endoderm	0.03	0.22	0.19	0.24	0.23	0.32
SOX17	Endoderm	0.00	0.02	0.03	0.01	0.00	0.02
SST	Endoderm	0.41	0.17	0.20	0.43	1.13	0.01

		Day 0	Day 3				
Target Name	Category	Non-transfected control	Non-transfected control	CHD7 DN1	CHD7 DN2	CHD7 DN1+DN2	mock
CXCL5	Self-renewal	2.66	1.08	1.36	2.29	1.63	1.51
DNMT3B	Self-renewal	0.78	0.49	0.55	0.56	0.57	0.66
HESX1	Self-renewal	0.66	2.87	2.55	1.85	1.77	2.24
IDO1	Self-renewal	0.90	1.26	1.46	1.06	1.36	1.31
LCK	Self-renewal	1.31	0.26	0.29	0.20	0.20	0.27
NANOG	Self-renewal	1.67	0.10	0.18	0.13	0.16	0.15
POU5F1	Self-renewal	1.05	0.09	0.21	0.14	0.19	0.14
SOX2	Self-renewal	0.64	2.20	2.48	1.52	1.81	2.04
TRIM22	Self-renewal	1.68	2.27	3.30	1.84	1.69	2.16

Fold change legend

fc > 100	Upregulated
10 < fc <= 100	
2 < fc <= 10	
0.5 <= fc <= 2	Comparable
0.1 <= fc < 0.5	
0.01 <= fc <0.1	
fc < 0.01	Downregulated
omitted	

Figure S9. Table depicting the expression of a 96-gene panel from *mock*-, *DN1*-, *DN2*- or *DN1+DN2*-transfected KhES-1 in an EB formation assay at day 0 or 3 determined by a qRT-PCR scorecard panel. This is a raw data before normalization for bar graphs in Figure 4C.

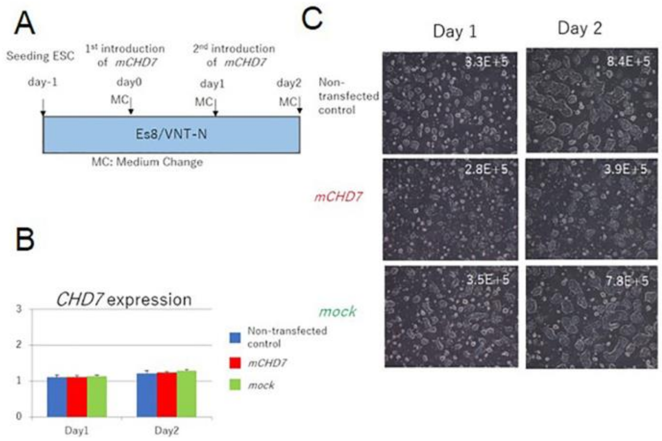

Figure S10. Introduction of *mCHD7* failed to generate *CHD7*-overexpressing ESCs in culture with Es8 medium.

A. Protocol for transfection with mRNA for *CHD7* isoform 2 (*mCHD7)* and cell culture.

B. Evaluation of *mCHD7* expression by qRT-PCR on days 1 and 2. The 3' PCR primer sets to detect all of CHD7 isoforms (isoform 1, 2 and X4) was used. *CHD7* gene expression was standardized according to the average *CHD7* expression in KhES-1 cells cultured with Es8 medium and measured independently 3 times. A representative result from 3 biological replicates is shown. (n=3 analytical replicates).

C. Cell counting and cell morphology of *CHD7*- or mock-transfected KhES-1 cells on days 1 and 2. The cell numbers in one well of a 6-well plate is appended in the upper right corner of the relevant photograph.

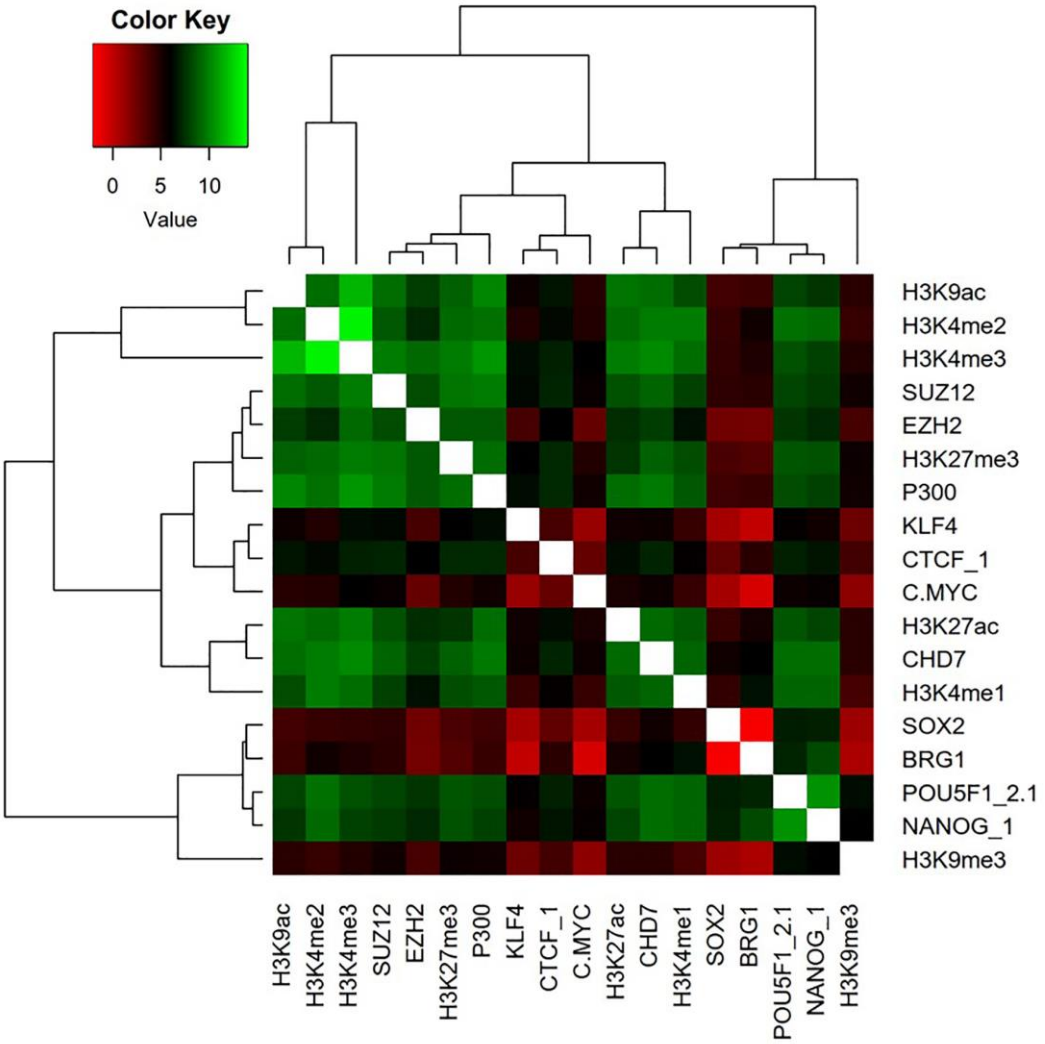

Figure S11. ChIP-seq 18 factors (H1) and BRG1 (H9). Heat map were obtained from a public ESC ChIP-seq database. The odds ratio representing the correlation between binding sites for each pair of factors was calculated. Green indicates high homology between factors and red indicates no high homology between factors.

Human ESC H1 or H9

Gene	Accession No.
CHD7	GSM1003473
P300	GSM1003513
SUZ12	GSM1003573
EZH2	GSM1003524
POU5F1	GSM803438
NANOG	GSM803437
CTCF	GSM803419
KLF4	GSM447584
C-MYC	GSM447585
SOX2	GSM456570
BRG1	GSM602297
H3K4me1	GSM733782
H3K4me2	GSM733670
H3K4me3	GSM733657
H3K9me3	GSM1003585
H3K27me3	GSM733748
H3K27ac	GSM733718
H3K9ac	GSM733773

Figure S12. List of accession numbers from the GEO public database
GSM accession numbers in the GEO database used for odds ratios mapping and gene expression profiles.

qPCR Primers

Gene	F/ R	seq (5'-3')
POU5F1	F	GAA ACC CAC ACT GCA GCA GA
POU5F1	R	TCG CTT GCC CTT CTG GCG
SOX2	F	GGG AAA TGG GAG GGG TGC AAA AGA GG
SOX2	R	TTG CGT GAG TGT GGA TGG GAT TGG TG
NANOG	F	CTC AGC TAC AAA CAG GTG AAG AC
NANOG	R	TCC CTG GTG GTA GGA AGA GTA AA
p300	F	TTG AAT GTA CAG AGT GCG GAA GA
p300	R	AAC AGC CAT CAC AGA CGA ATC C
CHD7	F	GGT TCC CAC ACT CGT GCA TA
CHD7	R	TGC GCC TCG GGA CAG A
CHD7 isoform 2	F	CCC ATG AAA GCA ATG AGT AAT CC
CHD7 isoform 2	R	TCC ATT GGT ATC CCA GCA CTT C
CHD7 Chromodomain	F	TGATGGACTTGGAACACAAAGTG
CHD7 Chromodomain	R	TGAAGGGAAGCGACTTGGTT
CHD7 sant slide	F	CAAACATGGCTATGAGAAGTACAACTC
CHD7 sant slide	R	CCGACTCGTTCCAGAAAGCA
GAPDH	F	CCA CTC CTC CAC CTT TGA CG
GAPDH	R	ATG AGG TCC ACC ACC CTG TT

ChIP Primers

Refseq No.	Gene	Primer
NM_203289.3	POU5F1	GPH1024786(-)01A
NM_024865.2	NANOG	GPH1002937(-)01A
NM_001429.3	p300	GPH1008986(-)01A
NM_001128844.1<br>NM_003072.3	BRG1	GPH1006548(-)01A
NM_015355.2	SUZ12	GPH1005657(-)01A
NM_152998.1<br>NM_004456.3	EZH2	GPH1025912(-)01A

Taq man PCR primer set 1

Refseq No.	Gene	Taqman probe
NM_017780<br>XM_011517553.2<br>XM_011517554.2<br>XM_011517555.2<br>XM_017013612.1<br>XM_017013613.1<br>XM_011517560	CHD7	Hs00215010_m1

Taq man PCR primer set 2

Refseq No.	Gene	P/F/ R	seq (5'-3')
NM_001316690	CHD7	P	TATGACTCAGAAACCGAAACAGAAACGACA
	CHD7	F	GCCCTTTCTAGAGAAACCAGTG
	CHD7	R	AGGCACCCTTTCTTCTCCTG

Taq man PCR primer set 3

Refseq No.	Gene	P/F/ R	seq (5'-3')
NM_017780<br>XM_011517553.2<br>XM_011517554.2<br>XM_011517555.2<br>XM_017013612.1<br>XM_017013613.1	CHD7	P	CACGGACGCTATAAACGCCAACTCACTG
	CHD7	F	GAATCTGCTTGTCTATGGTTGGG
	CHD7	R	AGGATGGTTCTGCAGATGGT

Figure S13. List of primer sets used for qRT-PCR.
